# Effectiveness of implementing an improvement cycle in the identification of critically ill patients

**DOI:** 10.1590/0034-7167-2021-0346

**Published:** 2022-07-18

**Authors:** Maria Solange Moreira de Lima, Kauanny Vitoria Gurgel dos Santos, Tâmara Taynah Medeiros da Silva, Joyce Karolayne dos Santos Dantas, Sara Cristina Matias de Araújo, Alyne Kelly de Oliveira Genuino, Daniele Vieira Dantas, Rodrigo Assis Neves Dantas

**Affiliations:** I Universidade Federal do Rio Grande do Norte. Natal, Rio Grande do Norte, Brazil

**Keywords:** Patient Safety, Patient Identification Systems, Health Care Quality, Total Quality Management, Nursing., Seguridad del Paciente, Sistemas de Identificación de Pacientes, Calidad de la Atención de Salud, Gestión de la Calidad Total, Enfermería., Segurança do Paciente, Sistemas de Identificação de Pacientes, Qualidade da Assistência à, Saúde, Melhoria da Qualidade, Enfermagem.

## Abstract

**Objective:**

To evaluate the effectiveness of implementing a quality improvement cycle in the process for identifying critically ill patients in an intensive care center.

**Methods:**

The implementation of an observational and interventional improvement cycle, using a before-and-after quasi-experimental design, with a quantitative approach, in an intensive care center. Seven criteria were developed to evaluate the quality of the identification process. The results of the intervention were subjected to statistical analysis.

**Results:**

The quality of the identification process showed significant improvement in the values referring to compliance with the conformities in the criteria evaluated. Statistical significance was observed in the evaluations of criteria C1, C2, C3, C4, and C6, with increased compliance values after the intervention.

**Final considerations:**

The efficacy of the improvement cycle in the quality of the patient identification process was evidenced. It was possible to involve and encourage the participation of the care team and improve organizational processes.

## RESUMO

**Objetivo:**

Avaliar a efetividade da implantação de um ciclo de melhoria da qualidade no processo de identificação do paciente crítico em um centro de cuidados intensivos**. Métodos:** Trata-se da implementação de ciclo de melhoria, observacional e de intervenção, utilizando desenho quase experimental, antes e depois, com abordagem quantitativa, em um centro de cuidados intensivos. Foram elaborados sete critérios para avaliação da qualidade do processo de identificação. Os resultados da intervenção foram submetidos à análise estatística.

**Resultados:**

A qualidade do processo de identificação apresentou melhoria significativa nos valores referentes ao cumprimento das conformidades nos critérios avaliados. Foi observada significância estatística nas avaliações dos critérios C1, C2, C3, C4 e C6, com aumento nos valores de cumprimento após a intervenção.

**Considerações finais:**

Evidenciou-se a eficácia do ciclo de melhoria na qualidade do processo de identificação do paciente. Foi possível envolver e estimular a participação da equipe assistencial e melhorar os processos organizacionais.

## RESUMEN

**Objetivo:**

Evaluar efectividad de implantación de un ciclo de mejoría de calidad en el proceso de identificación del paciente crítico en un centro de cuidados intensivos**. Métodos:** Implementación de ciclo de mejoría, observacional e intervención, utilizando diseño cuasi-experimental, antes y después, con abordaje cuantitativo, en un centro de cuidados intensivos. Elaborados siete criterios para evaluación de calidad del proceso de identificación. Los resultados de la intervención fueron sometidos al análisis estadístico.

**Resultados:**

La calidad del proceso de identificación presentó mejoría significativa en los valores referentes al cumplimiento de las conformidades en los criterios evaluados. Observada significación estadística en las evaluaciones de los criterios C1, C2, C3, C4 y C6, con aumento en los valores de cumplimiento posintervención.

**Consideraciones finales:**

Evidenciado la eficacia del ciclo de mejoría en la calidad del proceso de identificación del paciente. Fue posible involucrar y estimular la participación del equipo asistencial y mejorar los procesos organizacionales.

## INTRODUCTION

Patient safety has been getting more attention worldwide. Considered one of the aspects of health care quality, it is defined by the World Health Organization (WHO) as reducing the risk of damage related to health care, within the minimum acceptable level, being widely discussed and becoming essential for the development of actions that can ensure satisfactory and safe care^(1-2)^.

To improve care quality and minimize safety incidents, in April 2013, the Ministry of Health published Ordinance No. 529, which establishes the National Program for Patient Safety. This advocates proper patient identification as so to regulate the safety process in the care provided by health institutions and prevent possible damage^(3)^.

Mistakes in this process may occur from the moment of admission until the patient’s discharge, as some factors may increase the risks, such as the patient’s level of consciousness, bed or sector transfers, among other situations. The occurrence of failures due to lack of identification, or when it is performed incorrectly, may cause damages such as improper drug or blood product administration and performing of inadequate procedures^(3-5)^.

Correct patient identification and compliance with safety measures are essential to restore health, as well as to prevent and minimize possible complications during the patient’s stay in the institution. Thus, the proper identification has great relevance in care^(6)^. Patients in intensive care units (ICUs) are more susceptible to the occurrence of security incidents due to the instability of their clinical condition, in addition to the greater number of procedures to which they may be subjected^(7)^.

According to data from the bulletin of the Sistema Nacional de Vigilância Sanitária (SNVS) [National Health Surveillance System], between September 2019 and August 2020, there were more than 10 thousand patient identification failures^(8)^.

Therefore, in the pursue to improve the identification and minimize failures in care, it is essential to standardize correct identification, and to involve and educate professionals. The Cause-and-Effect Diagram and the brainstorming technique were applied by the Patient Safety Center together with the hospital team, which made it possible to identify the opportunities for improvement evidenced by the study. These efforts will help in the professional qualification on patient safety, allowing the mitigation of errors in health care. That said, such contributions justify this study’s advocacy for the implementation of the improvement cycle.

Based on this problematic, the following research questions emerged: How to implement an improvement cycle for the patient identification process in an intensive care center and what are the results after implementation?

## OBJECTIVE

To evaluate the effectiveness of implementing a quality improvement cycle in the process for identifying critically ill patients in an intensive care center.

## METHODS

### Ethical aspects

The study followed the ethical principles of research involving human beings, according to Resolution 466/12 of the National Health Council (NHC) and was approved by the Research Ethics Committee of the Onofre Lopes University Hospital (HUOL/UFRN) in 2019^(9)^. Patients and/or guardians signed an Informed Consent Form (ICF).

### Study design, period and location

The implementation of an observational and interventional improvement cycle, using a before-and-after quasi-experimental design, with a quantitative approach. Quasi-experimental studies are characterized by not having a randomized sample, which is influenced by the researchers involved in the process and are used in health care evaluation. Among the non-randomized studies, the before-and-after type design is included, in which an uncontrolled comparison is made between the frequencies of the results in two distinct moments of evaluation; or, still, it can be an action whose results are before and after its realization^(10)^.

The study was developed from November 2019 to December 2020, at Hospital Rio Grande (HRG), in the city of Natal, state of Rio Grande do Norte (RN). It is a private facility with about a thousand collaborators, 420 of whom are nurses. It has 194 beds, 56 of which make up the intensive care center (ICC), divided into five ICUs - 44 adult, 10 pediatric, and 2 for hemodialysis.

Improvement cycles are applied following previously determined steps to cyclically test implemented actions that should be monitored and evaluated^(11)^. The Standards for Quality Improvement Reporting Excellence 2.0 (SQUIRE) model and standards were followed, as it presents a structure and description of the standards used in the improvement cycles.

### Population or sample; inclusion and exclusion criteria

Service recipients are the patients who undergo hospital admission. Providers are all health professionals who provide care in the intensive care unit. The sample was carried out by convenience considering the total number of beds in the five ICUs of the ICC during the research period, with no inclusion or exclusion criteria, which totaled 52 patients. In addition, 160 health professionals who provide care in the intensive care unit were trained, being: 20 doctors, 20 nurses, 100 nursing technicians, and 20 physical therapists.

### Study protocol

The stages for applying the improvement cycle were as follows: 1) Identification and prioritization of the improvement opportunity; 2) Analysis of the quality problem; 3) Construction of the criteria to evaluate quality; 4) Planning of the study to evaluate the quality level; 5) Improvement intervention directed at the most problematic criteria; 6) Re-evaluation and registering of the improvement^(11)^. Regarding the execution period: Stages 1, 2, and 3 were carried out in November 2019; Stages 4 and 5, between the months of April and July 2020; and Stage 6, carried out from October to November 2020.

Thus, in Stage 1, an analysis of the opportunities for improvement was performed with the Patient Safety Center (PSC) team, through the brainstorming technique. Then, the nominal group technique and the problem prioritization matrix were applied - the latter presented as a priority the improvement in the patient identification process.

Within the prioritization matrix, the following problems were identified: improvement in the patient identification process, protocol for falls, organization of surgery scheduling, pharmacy medication dispensing, implementation of safe surgery protocols, and double checking the dispensing and administration of medications. For this, the following criteria were evaluated: if the problem affects many patients, if it represents a serious health risk, if the possible solution depends on internal efforts, and if it is a cheap solution.

In the second stage, after selecting the improvement opportunities, the possible causes for patient identification failure were found in order to proceed with the improvement planning. The following were used: brainstorming with the PSC members, composed of a physician, pharmacist, nurse, and nutritionist; and the cause-and-effect diagram (Ishikawa), to raise possible causes for the problem and then classify them. Aiming to better direct interventions, these causes were grouped into six categories: People, Processes, Equipment, Materials, Users, and Methods.

In the People category, the following was established: high staff turnover, lack of professional adherence, lack of continuous assessment by the sector team for replacement. In Processes: lack of a safety culture, lack of periodic education, lack of monitoring indicators. In Equipment: few printers to issue labels, low investment in new equipment, few computers available to the team. In Materials: lack of bedside identification, non-flexible wristband, permeable adhesive label. In Users: unawareness of information, patients remove their wristband, lack of orientation by the institution’s team. In Methods: outdated protocols, disorganization of medical records, insufficient number of personnel to carry out permanent health education and monitoring.

In the third stage, a construction of criteria to evaluate quality based on an instrument available at the service and on the Ministry of Health’s identification protocol was carried out. Seven quality criteria related to patient identification were developed, measured, and evaluated before and after the intervention, as shown in Chart 1.

**Chart 1:** Criteria for assessing the technical-scientific quality of patient identification, Natal, Rio Grande do Norte, Brazil, 2019

**CRITERIA**	**EXCEPTIONS**	**CLARIFICATIONS**
C1. Presence of an identification wristband	Patients with edema, injuries, limb restriction and/or amputation	
C2. Legible data on wristband		
C3. Wristband with accurate data		Full name, date of birth and/or medical record number
C4. Placement on the limb as per protocol	Swollen, injured, restricted and/or amputated limbs	Right upper limb
C5. Identification done on the cover of the medical chart containing at least two identifiers		No abbreviations.
C6. Identification done on all pages of the medical chart		Input the patient’s full name and at least one other identifier, such as registration number and/or date of birth.
C7. Bedside identification		Identification displayed in a manner visible to all, containing at least the full name and date of birth. For pediatrics, the name of the mother is required.

The criteria were evaluated as to the percentage of compliance, observing all patients admitted to the ICC in November 2019. With the data collected, it was possible to prepare a training plan for the team with the main requirements.

Data collection was carried out at the ICC, being divided into three moments: an initial assessment before the intervention, which occurred in November 2019; an additional collection, one month after the intervention, in October 2020; and, finally, the third collection, two months after the intervention, in November 2020. After collection, in the fourth stage of the study protocol, the data were analyzed and used for intervention planning focusing on the identified defects in quality. The fifth stage consisted of carrying out the intervention based on two strategic lines: organization of work processes and intervention with the team, as shown in Chart 2.

**Chart 2:** Organization of the permanent education intervention implemented after the first stage of data collection, Natal, Rio Grande do Norte, Brazil, 2019

**ORGANIZATION OF WORK PROCESSES**	**INTERVENTION WITH THE TEAM**
Updating the institution’s identification protocol; Review of the Standard Operating Protocol; Proposal for replacement of identification wristbands; Changing of the identification plates at bedside and reconfiguration of the layout of the displays; Issuing of identification plates via electronic medical record.	Permanent education, at alternate times, with the ICC nurses, exposing the adverse events related to the patient identification process; Standard Operating Protocol training with the nurses, who were then made responsible for training and implementing the process for the team.

In addition to relying on two strategic lines, the intervention was also structured in four phases: 1) recruitment of the professionals; 2) presentation and discussion of the institutional documents; 3) in-service training; and 4) resources for identification.

In the first phase, which consists of recruiting the professionals, five meetings were held with the ICC nurses, with expository dialogues about the importance of patient identification. The data from the first evaluation were presented, to serve as a starting problem-situation for the other phases.

The second phase pertains to the presentation and discussion of institutional documents, with the institutional protocol and a Standard Operating Protocol (SOP) on correct patient identification, which had been reviewed, being made available for reading and discussion.

The third phase refers to in-service training. In this context, and after the meetings, the nurses carried out a permanent education of their teams based on what had been previously discussed. The nurse of each shift used the SOP to discuss with the team. Thus, the actions occurred over a period of three weeks, in all shifts, to include the largest possible number of professionals.

Finally, the fourth and last phase deals with the identification resources. In parallel to the educational activities, a proposal was sent to management, aiming both to replace the identification wristbands, since the ones in use did not meet the requirements, and to acquire the displays for bedside identification for standardization. It is worth mentioning that the educational activities were done together with the institution’s Permanent Education Center (PEC).

In the sixth stage of the protocol, two re-evaluations were made after the intervention to verify whether the criteria under analysis were in conformity or not, the first in October 2020, and the second in November 2020. Thus, the following criteria were used: presence of an identification wristband, legible data on the wristband, wristband with correct data, placement on the limb according to protocol, identification on the cover of the medical chart containing at least two identifiers, identification on all sheets in the medical chart, and bedside identification. Direct observations of the patient, the bed, as well as the chart were also made.

### Analysis of results and statistics

A descriptive analysis of the variables was performed using absolute and relative (%) frequency distributions. In the evaluation of compliance by analyzed criteria, the chi-squared test (χ^***2***^) was applied, considering a significance level of 5%. To analyze the effect of the intervention, absolute and relative improvement values and statistical significance were estimated by the Z-test (p < 0.05). The non-compliance data of the assessments were analyzed using a before and after Pareto chart to visualize the improvement and prioritize quality criteria.

The Pareto Chart evaluates the non-compliance results using two vertical axes (the left, for the absolute number of non-compliance cases; and the right, for the corresponding relative frequencies calculated as a percentage in relation to the total number of non-compliance cases in the evaluation); while the horizontal axis originates a bar graph of the different criteria evaluated, numerically ordering from the most to the least frequent in non-compliance^(12)^.

## RESULTS

During the application of the first stage of the improvement cycle and identification of the main problems, brainstorming and the problem prioritization matrix were done with the PSC team to determine the priority for improvement, which, in this case, was patient identification. For the second stage, involving problem analysis and quality, brainstorming and the cause-and-effect diagram (Ishikawa) were adopted to raise the causes of the problems and classify them into six categories: People, Processes, Equipment, Materials, Users, and Methods.

Aiming to analyze the effectiveness of these actions in the steps of the improvement cycle in patient identification, it was necessary to implement the seven aforementioned evaluation criteria, before and after application, in order to obtain satisfactory results and assist in improving the quality of services.

The first evaluation showed variable levels of compliance with the criteria. Among the 52 patients evaluated, the criteria that presented the lowest compliance were C2 and C3. Criterion C4 stands out as well, with only 17% compliance. Regarding identification in the medical chart, criterion C6 achieved only 25% compliance, as shown in Table 1.

**Table 1:** Initial assessment of compliance and non-compliance by analyzed criteria, Natal, Rio Grande do Norte, Brazil, 2019

**Criterion**	**Initial assessment**	
	**Compliant**	**Non-compliant**
C1	50.00% (n = 26)	50.00% (n = 26)
C2	32.69% (n = 17)	67.31% (n = 35)
C3	32.69% (n = 17)	67.31% (n = 35)
C4	17.31% (n = 9)	82.69% (n = 43)
C5	98.08% (n = 51)	1.92% (n = 1)
C6	25.00% (n = 13)	75.00% (n = 39)
C7	59.62% (n = 31)	40.38% (n = 21)
Total	100.00% (n = 52)	

Regarding the fourth stage, it was observed the need to evaluate the criteria at three moments: the first, before the intervention in November 2019; the second, after the intervention in October 2020; and the third, two months after the collection in November 2020. After these reassessments, the quality of the identification process showed significant improvement in the values referring to compliance with conformities, when comparing the pre-intervention and post-intervention results, as evidenced in Table 2.

**Table 2:** Evaluation and re-evaluation of compliance with the conformities and nonconformities by analyzed criteria, Hospital Rio Grande, Natal, Rio Grande do Norte, Brazil, 2019

**Criterion**	**Initial assessment**		**First re-evaluation**		**Second re-evaluation**		***p* value**
	**Compliant**	**Non-compliant**	**Compliant**	**Non-compliant**	**Compliant**	**Non-compliant**	
C1	50.00% (n = 26)	50.00% (n = 26)	85.71% (n = 42)	14.29% (n = 7)	73.08% (n = 38)	26.92% (n = 14)	**< 0.001**
C2	32.69% (n = 17)	67.31% (n = 35)	73.47% (n = 36)	26.53% (n = 13)	59.62% (n = 31)	40.38% (n = 21)	**< 0.001**
C3	32.69% (n = 17)	67.31% (n = 35)	73.47% (n = 36)	26.53% (n = 13)	59.62% (n = 31)	40.38% (n = 21)	**< 0.001**
C4	17.31% (n = 9)	82.69% (n = 43)	63.27% (n = 31)	36.73% (n = 18)	53.85% (n = 28)	46.15% (n = 24)	**< 0.001**
C5	98.08% (n = 51)	1.92% (n = 1)	100.00% (n = 49)	-	98.08% (n = 51)	1.92% (n = 1)	0.620
C6	25.00% (n = 13)	75.00% (n = 39)	73.47% (n = 36)	26.53% (n = 13)	69.23% (n = 36)	30.77% (n = 16)	**< 0.001**
C7	59.62% (n = 31)	40.38% (n = 21)	77.55% (n = 38)	22.45% (n = 11)	73.08% (n = 38)	26.92% (n = 14)	0.121
Total	100.00% (n = 52)		100.00% (n = 49)		100.00% (n = 52)		

When applying the chi-squared test, there was evidence of a statistically significant difference in the evaluations in criteria C1, C2, C3, C4, and C6. A higher percentage of compliance can be observed in the respective criteria in the first and second re-evaluation, compared to the initial evaluation. Criterion C5 already showed a high compliance since the initial evaluation and remained as such in the others, so there was no statistical significance.

**Figure 1:**
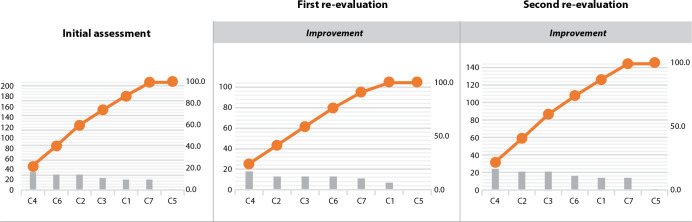
Pareto diagram showing the frequency of non-compliance of quality criteria, Natal, Rio Grande do Norte, Brazil, 2020

The fifth stage, consisting of the improvement intervention targeting the most problematic criteria, was performed by evaluating the improvement opportunities identified after the first evaluation. It was observed that the vital points were the placement of the wristband on the limb according to the institutional protocol (C4), identification on all pages of the medical chart (C6), legible and correct data in the identification wristband (criteria C2 and C3, respectively), accumulating a frequency of 76% of all quality defects, as presented in Figure 1.

The sixth stage consisted in the re-evaluation and recording of improvements. For this, based on the sum of failures of all criteria used for quality assessment in patient identification, it was possible to observe 200 non-compliances in the initial assessment and 75 in the first re-evaluation, which represents a reduction of 125 quality defects. And, although there was an increase to 111 in the second re-evaluation, a relevant improvement is perceived. The demonstration of the results allows us to highlight a significant improvement in the criteria when comparing the initial evaluation with the two re-evaluations. It is noteworthy that the first re-evaluation presented higher criteria conformities, to the detriment of the second evaluation, which indicates the need for continuity in team building.

## DISCUSSION

Regarding criterion C1 (presence of an identification wristband), it was identified in the initial assessment that 50% of patients had a wristband, but only 32% had legible and correct data (Criteria 2 and 3). After the intervention, in the first re-evaluation, 85% of the patients had a wristband, and 73% of them had legible and correct data.

A study carried out in three ICUs of a large hospital in Bahia showed that only 59% of the patients were wearing an identification wristband, and in only 37.6% of them the data was legible, results similar to those found in the initial assessment^(7)^. A study in Turkey showed a rate of 84.5% of patients identified with a wristband^(13)^.

The permanence of this identification goes from the admission to the discharge of the user due to the risk of several assistance errors during his/her hospitalization period, such as: medication or hemotransfusion errors, performance of diagnostic tests or procedures, be it either on the patient or in the wrong place. The instability of critically ill patients in the ICU and the number of procedures and devices to which they are submitted increase the chance of adverse events that may lead to loss of confidence, patient disability, or death^(14)^.

Thus, failures in patient identification affect health care, favoring the occurrence of deaths, sequelae, negligence, in addition to patient suffering, factors that cause the loss of patient confidence in the service^(15)^. The identification should reach 100% of patients, since it is a step that precedes all the care provided by the health team. In this sense, the present study obtained better results only after the intervention, and this indicates that the educational plan is a factor in mitigating incidents related to patient identification^(16)^.

A study evaluating the perception of patients regarding the identification wristband showed that no professional had put it on and others stated that they received it upon admission, but kept it in a drawer, as they did not realize the importance of the device. Thus, the health care team has a crucial role in the identification and surveillance of the conservation of the wristband^(17)^.

The absence of patient identification is attributed to gaps related to the patient’s understanding of the need for the wristband and professionals working to maintain and follow patient safety routines. There are factors related to the patient’s condition, such as edema and limb amputations, excess of devices, and low level of consciousness^(17-18)^. In this line, the patient must be oriented about the importance of using the identification wristbands, and it is also necessary to recommend not removing them and to check if they are illegible or worn out^(13)^.

The literature highlights that failures in the process of patient identification can generate possibly severe damage, among which 9% would be temporary or permanent. Furthermore, it reveals that in the United States, on average, 850 patients undergo blood transfusion not related to their treatment, causing 3% of them to die for this reason^(4)^.

The difficulty of identification in the pediatric ICU, due to the limited flexibility of the wristbands, stands out as a weakness for process adjustment. When evaluating the data per ICU, it was not possible to observe improvement, as in the first assessment 20% of the patients had identification wristbands, and in the second and third assessment, they reached 30% and 20%, respectively. After the presentation of the data to management, specific wristbands were acquired for this public.

In a study conducted in the pediatric ICU of three hospitals in southern Brazil, it was found that there was no identification by wristband, and 98% of the beds were identified by bedside signs. Failures in the identification in the medical record were also found, a result similar to this study, demonstrating the safety vulnerability for the patient^(19)^.

Regarding criteria C2 and C3, the identification labels of the wristbands were erased, which made it impossible to read the data and confirm them, thus reaching only 32% of compliance. In this sense, it should contain at least two relevant pieces of information: full name, date of birth, and/or registration number^(20)^. Differently from the data found, a research conducted in a university hospital in the South Region found that only 0.81% of the wristbands showed material damage that prevented the reading and guarantee of data^(16)^.

The professionals’ lack of knowledge about the institutional protocol and non-adherence to identification practices were noted, as the criteria with the highest non-compliance were not placing the identification wristband on the limb according to the institutional protocol (C4) and non-identification on the medical record sheets (C6), corresponding to 21.5% and 19.5%, respectively. Data from a study conducted in three hospitals in Rio Grande do Norte to verify patient identification in the medical record attested to the results found, indicating fragility in the process^(4)^.

As for criterion C7 (Use of identification signs at bedside), the values in the pre-intervention assessment were 59% of compliance rate. However, there was no standardization for these signs in the institution, and each ICC unit had developed its own model individually. After the intervention, the rate increased to 77%. In a study carried out in six ICUs in São Paulo, 99.47% of the beds had identification, a number higher than in this study^(21)^. However, bedside identification should not be the only way to identify the patient, and should occur sequentially to the identification wristband check, according to the Ministry of Health’s protocol^(1)^.

Continuing education aids patient safety. Research conducted in Ireland adopted an educational program aimed at safety in medication management, consisting of three phases. In the first phase, the organization’s behaviors and policies for medication use were analyzed. In the second phase, interviews were conducted with patient safety experts to refine the knowledge of health professionals. The third phase was a case study and discussion. Overall, the initiative trained the clinical reasoning of the professionals, reducing errors in medication application^(22)^.

Education is necessary for all healthcare professionals in order to mitigate adverse events and improve their indicators. As nurses are fundamental in the construction of safe practices, they need to have a theoretical basis to develop their actions, reduce the chances of these events, and provide guidance about the importance and usefulness of the identification wristband for patient safety^(23)^. Interdisciplinarity is important for the effectiveness of continuing education in health, but there is still professional resistance, since work fragmentation hinders interdisciplinary action, reflecting negatively on the safety culture^(24)^.

It is noteworthy that the period between the request and the availability of bedside identification displays by the institution, the replacement of identification wristbands, as well as the lack of unanimous adherence to the new routines established by the ICU professionals, constituted obstacles for the advancement of the research.

### Study limitations

This study is limited to presenting only the reality of a private hospital in the state of Rio Grande do Norte. Furthermore, the data collection time period available was of only one year with a sample selected by convenience, which may limit data generalization. Finally, it was difficult to find studies and publications with criteria to identify patients in the intensive care setting.

### Contributions to the field of Nursing, Health, or Public Policies

It is believed that studies such as this one can contribute to the improvement of the user identification process during hospital stay, offering relevant information to minimize failures in the provision of care and the occurrence of incidents, as well as the implementation of the safety culture in health services.

## FINAL CONSIDERATIONS

The quality improvement cycle proved to be effective in improving the process of patient identification. The initial assessment showed weaknesses and the need for an intervention focused on education and restructuring of institutional processes related to patient identification. We highlight the strengthening of the Patient Safety Center and the Permanent Education Center, which were able to improve the process and sensitize the team.

With this study, it was possible to improve the process of patient identification in this institution, showing the effectiveness of the action. In this sense, stipulating routines for safe care requires educational investments, which will help in the advancement and formation of the safety culture.

The educational activities were essential to disseminate the institutional protocol and sensitize professionals to implement and adhere to the process. Leadership support was also essential, especially the nurses, who were responsible for discussing the SOP with their team. Despite the noticeable improvement, for the sustainability of the process it is necessary to perform periodic monitoring in order to maintain this improvement and develop weak points.

## SUPPLEMENTARY MATERIAL

This manuscript is the result of a master’s thesis linked to the Postgraduate Program in Quality Management in Health Services at the Federal University of Rio Grande do Norte (PPG QualiSaúde/UFRN) and has been added to the institutional repository, accessible through: https://repositorio.ufrn.br/handle/123456789/32707
