# Supplementary material for: Effectiveness of implementing an improvement cycle in the identification of critically ill patients
Source: Rev Bras Enferm. 2022 Jul 18;75(6):e20210346. doi: 10.1590/0034-7167-2021-0346 (PMC9728848; doi:10.1590/0034-7167-2021-0346)
Supplement: Supplementary file 1 [file 0034-7167-reben-75-06-e20210346-sup01.pdf]

UNIVERSIDADE FEDERAL DO RIO GRANDE DO NORTE
CENTRO DE CIÊNCIAS DA SAÚDE
MESTRADO PROFISSIONAL GESTÃO DA QUALIDADE EM SERVIÇOS DE SAÚDE

IDENTIFICAÇÃO DO PACIENTE EM UM CENTRO DE CUIDADOS INTENSIVOS: aplicação de um ciclo de melhoria

MARIA SOLANGE MOREIRA DE LIMA

NATAL/RN
2021

Universidade Federal do Rio Grande do Norte - UFRN
Sistema de Bibliotecas - SISBI
Catalogação de Publicação na Fonte. UFRN - Biblioteca Setorial do Centro Ciências da Saúde - CCS

Lima, Maria Solange Moreira de.
Identificação do paciente em um centro de cuidados intensivos: aplicação de um ciclo de melhoria / Maria Solange Moreira de Lima. - 2021.
53f.: il.

Dissertação (Mestrado Profissional em Gestão da Qualidade em Serviços de Saúde) - Universidade Federal do Rio Grande do Norte, Centro de Ciências da Saúde, Programa de Pós-Graduação em Gestão da Qualidade em Serviços de Saúde. Natal, RN, 2021.
Orientador: Rodrigo Assis Neves Dantas.
Coorientadora: Daniele Vieira Dantas.

1. Segurança do paciente - Dissertação. 2. Sistemas de identificação de pacientes - Dissertação. 3. Qualidade da assistência à saúde - Dissertação. I. Dantas, Rodrigo Assis Neves. II. Dantas, Daniele Vieira. III. Título.

RN/UF/BS-CCS CDU 614.253.83

UNIVERSIDADE FEDERAL DO RIO GRANDE DO NORTE
CENTRO DE CIÊNCIAS DA SAÚDE
MESTRADO PROFISSIONAL GESTÃO DA QUALIDADE EM SERVIÇOS DE SAÚDE

IDENTIFICAÇÃO DO PACIENTE EM UM CENTRO DE CUIDADOS INTENSIVOS: aplicação de um ciclo de melhoria

Trabalho de Conclusão de Mestrado apresentado ao Programa de Pós-Graduação Mestrado Profissional Gestão da Qualidade em Serviços de Saúde, do Centro de Ciências da Saúde, da Universidade Federal do Rio Grande do Norte, como requisito para obtenção do título de Mestre em Gestão da Qualidade em Serviços de Saúde.

Maria Solange Moreira de Lima

Orientador: Prof. Dr. Rodrigo Assis Neves Dantas
Coorientadora: Prof.ª Dra. Daniele Vieira Dantas

NATAL/RN
2021

IDENTIFICAÇÃO DO PACIENTE EM UM CENTRO DE CUIDADOS INTENSIVOS: aplicação de um ciclo de melhoria

BANCA EXAMINADORA

Presidente da banca: Professor Dr. Rodrigo Assis Neves Dantas (Universidade Federal do Rio Grande do Norte – UFRN)

Professora Dra. Ana Elza Oliveira de Mendonça (Universidade Federal do Rio Grande do Norte – UFRN)

Professora Dra. Isabel Karolyne Fernandes Costa (Hospital Universitário Onofre Lopes – HUOL)

NATAL/RN
2021

DEDICATÓRIA

*Aos meus pais **Francisca e Expedito**, por acreditarem em mim, me apoiarem e serem a razão da minha vida.*

*Aos meus irmãos **José, Sérgio, Paulo, Francisco** (in memoriam) e **Alex** por serem os melhores irmãos que eu poderia ter.*

Aos meus amigos, por todo o amor, incentivo e compreensão, cujo apoio foi decisivo para a chegar até aqui.

Aos meus pacientes por serem inspiração e ensinamento.

AGRADECIMENTOS

A Deus, por me proporcionar esta oportunidade e me dar forças para seguir em frente, apesar de todas as dificuldades.

Ao meu orientador e amigo, Professor Rodrigo, por todo o incentivo, paciência e compreensão desde 2013 até hoje e por acreditar em mim, mesmo quando até eu não acreditei.

À minha família, por ser a minha base e me incentivar a ser uma pessoa melhor todos os dias.

Ao Hospital Rio Grande (HRG), em especial ao meu gerente Marcelo, enfermeira Karine e Dr. Luiz Roberto, por acreditarem no meu trabalho e possibilitarem a realização desta pesquisa.

À equipe do Centro de Cuidados Intensivos do HRG, que contribuiu para o alcance das melhorias.

Às queridas Caroline Schon, Alyne, Sara, Sara Cynthia, Joyce e Sabrina, que me apoiaram e me ajudaram a construir este trabalho.

Aos amigos do mestrado, com quem dividi angústias e alegrias, e pude construir uma relação singular, em especial a Sabrina, Raquel, Joyce, Fernanda e Rafael, apoiadores nos momentos mais difíceis e seres humanos ímpares.

A Kalyane, Andrea e Morgana, por serem minhas irmãs de coração, me apoiarem e acreditarem em mim.

Aos meus amigos, que me incentivaram e apoiaram em todos os momentos.

A Lucas, por ter tornado esta caminhada mais leve.

Minha gratidão e apreço a todos aqueles que, direta ou indiretamente, contribuíram para que esta dissertação se tornasse uma realidade.

SUMÁRIO

Dedicatória.. IV

Agradecimentos.. V

Lista de tabelas.. VII

Lista de figuras.. VIII

Lista de quadros.. IX

Resumo... X

Abstract... XI

Resumen... XII

APRESENTAÇÃO... XIII

1 ANEXAÇÃO DO ARTIGO... 14

1. 1 Introdução.. 15

1. 2 Metodologia.. 16

1. 3 Resultados 24

1. 4 Discussão.. 28

1. 5 Conclusão... 32

REFERÊNCIAS.. 33

2 APÊNDICES.. 37

2.1 Apêndice 1 – Matriz de priorização de problemas - HRG, Natal/Rio Grande do Norte, 2019.. 38

2.2 Apêndice 2 – Diagrama de causa e efeito aplicado à identificação dos pacientes do HRG, Natal/Rio Grande do Norte, 2019... 39

2.3 Apêndice 3 – Instrumento de Coleta de dados.. 40

2.4. Apêndice 4 – Termo de Consentimento Livre e Esclarecido para profissionais... 41

2.5. Apêndice 5 – Termo de Consentimento Livre e Esclarecido para maiores de idade.. 44

2.6. Apêndice 6 – Termo de Consentimento Livre e Esclarecido para responsáveis 47

3 ANEXOS... 50

3. 1 Parecer do Comitê de Ética em Pesquisa 51

3. 2 Carta de Anuência.. 53

Lista de Tabelas

**Tabela 1.**	Avaliação inicial do cumprimento de conformidade e não conformidade por critério analisado, Natal/Rio Grande do Norte, 2019	24
**Tabela 2.**	Avaliação e reavaliação do cumprimento das conformidades e não conformidades por critério analisado, Natal/Rio Grande do Norte, 2019 a 2020	24

Lista de Figuras

Figura 1. Diagrama de causa e efeito aplicado à identificação dos pacientes, Natal/Rio Grande do Norte, 2019 39

Figura 2. Diagrama de Pareto evidenciando a frequência de não conformidades, Natal/Rio Grande do Norte, 2020 27

Lista de Quadros

Quadro 1. Matriz de priorização de problemas, Natal/Rio Grande do Norte, 2019 38

Quadro 2. Critérios para avaliar a qualidade técnico-científica da identificação dos pacientes, Natal/Rio Grande do Norte, 2019 19

Quadro 3. Organização da intervenção de educação permanente a ser implementada após a primeira etapa da coleta de dados, Natal/Rio Grande do Norte, 2019 22

Resumo

Introdução: Definida pela Organização Mundial da Saúde (OMS), a identificação do paciente é uma ação que tem como objetivo regulamentar o processo de segurança na assistência prestada pelas instituições de saúde, bem como minimizar a possibilidade de danos e, ainda, assegurar que determinado cuidado, procedimento ou tratamento seja destinado a ele. **Objetivo:** Avaliar o efeito da implantação de um ciclo de melhoria da qualidade no processo de identificação do paciente. **Metodologia:** Trata-se da implementação de um ciclo de melhoria interno, observacional e de intervenção, utilizando um desenho quase experimental, antes e depois, com abordagem quantitativa, em um Centro de Cuidados Intensivos. Foram elaborados sete critérios para avaliação da qualidade do processo de identificação. Os resultados da intervenção foram submetidos à análise estatística e apresentados em gráfico de Pareto, para demonstrar o nível de melhoria. **Resultados:** A qualidade do processo de identificação apresentou melhoria significativa nos valores referentes ao cumprimento das conformidades nos critérios avaliados. Foi observada significância estatística ($p < 0,001$) nas avaliações dos critérios C1, C2, C3, C4 e C6, com aumento nos valores de cumprimento após a intervenção do ciclo de melhoria. **Conclusões:** O presente estudo evidenciou que o ciclo de melhoria realizado com o objetivo de melhorar a qualidade do processo de identificação do paciente mostrou-se efetivo. Foi possível envolver as partes interessadas, estimular a participação da equipe assistencial e melhorar os processos organizacionais relacionados à temática.

Descritores: Segurança do paciente; Sistemas de identificação de pacientes; Qualidade da assistência à saúde.

Abstract

Introduction: Defined by the World Health Organization (WHO), the patient identification is an action that aims to regulate the safety process in care provided by health institutions, as well as to minimize the possibility of damage and to ensure that a certain care, procedure or treatment is intended for him. **Objective:** Evaluate the effect of implementing a quality improvement cycle on the patient identification process. **Methodology**: Involves the implementation of an internal, observational and intervention improvement cycle, using a almost experimental design, before and after, with a quantitative approach, in an Intensive Care Center. Were developed seven criteria to assess the quality of identification process. The results of intervention were subjected to statistical analysis and presented in Pareto graph, to demonstrate the level of improvement. **Results:** The quality of the identification process showed a significant improvement in the values referring to fulfillment of conformities in evaluated criteria. Statistical significance ($p < 0.001$) was observed in the assessments of criteria C1, C2, C3, C4 and C6, with an increase in compliance values after the intervention of improvement cycle. **Conclusions:** The present study showed that the improvement cycle realized with the aim of improving the quality of patient identification process proved to be effective. It was possible to involve the interested parties, to stimulate the participation of assistance team and improve the organizational processes related to the theme.

Keywords: Patient safety; Patient identification systems; Quality of health care.

Resumen

Introducción: Definida por la Organización Mundial de la Salud (OMS), la identificación del paciente es una acción que tiene como meta reglamentar el proceso de seguridad en asistencia a las instituciones de salud, así como minimizar la posibilidad de daño, además de asegurar que un determinado cuidado, procedimiento o tratamiento sea destinado a él. **Objetivo**: Evaluar el efecto de implementación de un ciclo de mejora de la calidad en el proceso de identificación de pacientes. **Metodología**: Se refiere a la implementación de un ciclo de mejoramiento interno, observacional y de intervención, utilizando un diseño casi experimental, antes y después, con enfoque cuantitativo, en un Centro de Cuidados Intensivos. Se desarrollaron siete criterios para evaluar la calidad del proceso de identificación. Los resultados de la intervención se sometieron a análisis estadístico y se presentaron en un gráfico de Pareto, para demostrar el nivel de mejora. **Resultados**: La calidad del proceso de identificación presentó una mejora significativa en los valores referentes al cumplimiento de las conformidades en los criterios evaluados. Se observó significación estadística ($p < 0{,}001$) en las evaluaciones de los criterios C1, C2, C3, C4 y C6, con un aumento en los valores de cumplimiento tras la intervención del ciclo de mejora. **Conclusiones**: El presente estudio mostró que el ciclo de mejora realizado con el objetivo de mejorar la calidad del proceso de identificación de pacientes resultó ser efectivo. Se logró involucrar a las partes interesadas, incentivar la participación del equipo de asistencia y mejorar los procesos organizacionales que se relacionan al tema.

Descriptores: Seguridad del paciente; Sistemas de identificación de pacientes; Calidad de la asistencia a la salud.

APRESENTAÇÃO

A estrutura do presente Trabalho de Conclusão de Mestrado segue um modelo próprio de disposição, recomendado e proposto pelo Programa de Pós-Graduação Mestrado Profissional Gestão da Qualidade em Serviços de Saúde da Universidade Federal do Rio Grande do Norte (PPQualiSaúde/UFRN). O trabalho de Conclusão de Mestrado encontra-se no formato de um artigo científico. Ainda não segue normas específicas de um periódico, pois haverá escolha da revista mais adequada e que acolha a temática, para fins de submissão. Assim, as referências bibliográficas encontram-se dispostas de acordo com as normas da Associação Brasileira de Normas Técnicas (ABNT).

A primeira seção, Introdução, apresenta brevemente o objeto da pesquisa, bem como o objetivo desta investigação no formato de artigo científico.

A seção seguinte, intitulada Metodologia, tem como tarefa sistematizar os procedimentos metodológicos percorridos para a execução do trabalho.

Na sequência, a seção Resultados apresenta os resultados relevantes encontrados através do desenvolvimento do trabalho.

A Discussão do artigo apresenta um diálogo entre os resultados do presente trabalho encontrados e a literatura científica.

E as Conclusões encerram o artigo científico, destacando os principais resultados e apontando para novas investigações.

1 ANEXAÇÃO DO ARTIGO

IDENTIFICAÇÃO DO PACIENTE EM UM CENTRO DE CUIDADOS INTENSIVOS: aplicação de um ciclo de melhoria

1.1 Introdução

A segurança do paciente tem ganhado cada vez mais evidência em todo o mundo. Considerada uma das dimensões da qualidade em saúde, é definida pela Organização Mundial da Saúde (OMS) como a redução do risco de danos relacionados à assistência em saúde, dentro do mínimo aceitável, sendo amplamente debatida e tornando-se imprescindível para o desenvolvimento de ações que possam garantir uma assistência satisfatória e segura (LEMOS; CUNHA, 2017; SAFETY, 2009).

Com o propósito de melhorar o nível de qualidade no processo assistencial e minimizar os incidentes de segurança, em abril de 2013, o Estado brasileiro publicou, por meio do Ministério da Saúde, a Portaria nº 529, que institui o Programa Nacional de Segurança do Paciente (BRASIL, 2013).

Ressalte-se que a primeira meta do Programa, definida inicialmente pela OMS, preconiza a adequada identificação do paciente, ação que tem como objetivo regulamentar o processo de segurança na assistência prestada pelas instituições de saúde, bem como minimizar a possibilidade de danos, assim como assegurar que determinado cuidado, procedimento ou tratamento seja destinado a ele. O erro nesse processo pode ocorrer desde o momento da admissão até a hora da alta do paciente, período no qual alguns fatores acabam potencializando ainda mais os riscos, tais como: nível de consciência do paciente, transferências de leitos ou setor, dentre outras situações. A ocorrência de falhas quando não há identificação, ou esta é realizada de maneira incorreta, pode acarretar danos como administração imprópria de medicamentos, hemocomponentes e até mesmo a realização de procedimentos inadequados (ALVES *et al.,* 2018; BRASIL, 2013; GIRARDI *et al.,* 2018).

A correta identificação do paciente e a realização das medidas de segurança no processo do cuidado mostram-se essenciais para que haja restabelecimento da saúde, bem como para evitar e/ou minimizar possíveis intercorrências durante sua permanência na instituição. Dessa forma, o ato da adequada identificação ganha proporções relevantes no conjunto das ações de cuidados (MACEDO *et al.,* 2017).

Pacientes que se encontram internados em Unidades de Terapia Intensiva (UTI) são mais suscetíveis à ocorrência de incidentes de segurança, fato já evidenciado por estudos, devido à condição clínica do paciente, com potencial alteração do nível de consciência, além do maior número de procedimentos a que estes podem ser submetidos (MACEDO *et al.,* 2017; TRINDADE *et al.,* 2019).

Segundo dados disponíveis em boletim do Sistema Nacional de Vigilância Sanitária (SNVS), no período de setembro de 2019 a agosto de 2020, foram registradas pelos prestadores de serviços de saúde mais de 150.000 notificações de incidentes relacionados à assistência. Vale notar que, do total das notificações registradas, mais de 10.000 foram sobre falhas na identificação do paciente (BRASIL, 2020).

Portanto, na busca pela melhoria do processo de identificação segura e minimização das falhas em todo o processo assistencial, padronizar a identificação de maneira correta, envolver e educar os profissionais que lidam com o gerenciamento do cuidado são imprescindíveis. Para que isso ocorra, é preciso traçar estratégias e oferecer educação permanente com o intuito de sensibilizar os profissionais acerca da importância da identificação segura em todo o processo do cuidado. Tais esforços contribuirão para a qualificação dos profissionais sobre segurança do paciente e a implementação da cultura de segurança, fazendo com que haja uma diminuição nos erros relacionados à assistência à saúde.

Nesse contexto e com base nessa problemática, surgiram as seguintes questões de pesquisa: Como implementar um ciclo de melhoria para o processo de identificação do paciente e quais os resultados após a implementação? Dessa maneira, o presente estudo tem como objetivo avaliar o efeito da implantação de um ciclo de melhoria da qualidade no processo de identificação do paciente.

1. 2 Metodologia

1.2.1 Tipo de estudo

Este estudo trata da implementação de um ciclo de melhoria interno, observacional e de intervenção, utilizando um desenho quase experimental, antes e depois, com abordagem quantitativa, tendo sido desenvolvido no período de novembro de 2019 a dezembro de 2020, no Hospital Rio Grande (HRG), Natal, no Rio Grande do Norte.

O estudo com delineamento quase experimental do tipo antes e depois é caracterizado pela não randomização da amostra para a implementação da intervenção (NEDEL; SILVEIRA, 2016).

Os Ciclos de Melhoria da Qualidade estão compreendidos entre as três atividades de gestão dos serviços de saúde, acompanhados do planejamento e monitoramento. Eles têm o objetivo de esclarecer e solucionar problemas ou oportunidades de melhoria. Seu início é motivado por um problema e orientado para um objetivo focado. Assim, os ciclos de melhoria são aplicados permitindo testar ciclicamente ações implementadas que são monitoradas através do controle estatístico (SATURNO, 2015).

De modo objetivo, a sequência cíclica das atividades para um Ciclo de Melhoria da Qualidade é composta pela identificação e priorização da oportunidade de melhoria, análise do problema de qualidade, avaliação, intervenção e reavaliação (SATURNO, 2015; PORTELA *et al.,* 2015).

O estudo tem como dimensão a qualidade técnico-científica e os tipos de dados são de processo. Os receptores do serviço são os pacientes que se submetem a internação hospitalar. Os provedores são todos os profissionais de saúde que prestam assistência na unidade de terapia intensiva, dentre eles, médicos(as), enfermeiros(as), técnicos(as) de enfermagem, fisioterapeutas, farmacêuticos e nutricionistas da instituição.

O estudo seguiu o modelo e normas adotadas pelo *Standards for Quality Improvement Reporting Excellence* 2.0 (SQUIRE) por apresentar estrutura e descrição dos padrões utilizados nos ciclos de melhoria.

1. 2. 2 Contexto e Local do estudo

A pesquisa foi desenvolvida no HRG, localizado na cidade do Natal, no Estado do Rio Grande do Norte (RN). Esse hospital apresenta cerca de 1000 colaboradores, sendo 420 da Enfermagem. Dispõe de 194 leitos; destes, 54 compõem o Centro de Cuidados Intensivos (CCI), dividido em cinco Unidades de Terapia Intensiva (UTI), sendo 44 adultos e 10 pediátricos, e dois exclusivos para realização de hemodiálise. Com essa capacidade, atende as diversas especialidades, como: clínica médica, cirurgia geral, cardiologia, gastroenterologia, nefrologia, urologia, oncologia, hematologia, transplante de medula óssea e ortopedia. Possui também serviço de

imagem e de métodos gráficos com hemodinâmica, tomografia, ultrassonografia e ecocardiograma.

No ano de 2019, o hospital realizou cerca de 33 mil atendimentos no pronto atendimento, mais de 2000 mil procedimentos na unidade de hemodinâmica e cerca de 9000 cirurgias eletivas e de emergência no centro cirúrgico, para atender pacientes da saúde suplementar e do Sistema Único de Saúde (SUS) (RIO GRANDE DO NORTE, 2020).

1. 2. 3 População e amostra

A amostra foi realizada por conveniência, considerando o número total de vagas das cincos unidades de terapia intensiva do Centro de Cuidados Intensivos do HRG, totalizando um número de 52 pacientes.

1.2.4 Etapas do estudo

Este estudo seguiu a estratégia de aplicação de um ciclo de melhoria, sendo desenvolvido em seis etapas, a saber: 1) Identificação e priorização da oportunidade de melhoria; 2) Análise do problema de qualidade; 3) Construção dos critérios para avaliar a qualidade; 4) Planejamento do estudo para avaliar o nível de qualidade; 5) Intervenção de melhoria dirigida aos critérios mais problemáticos; 6) Reavaliação e registro da melhoria.

Etapa 1: Identificação e priorização da oportunidade de melhoria

Na primeira etapa do estudo, para identificação e priorização do problema de qualidade a ser selecionado, foi realizada uma análise das oportunidades de melhoria junto à equipe do Núcleo de Segurança do Paciente, por meio da técnica de chuva de ideias, na qual foram elencadas seis oportunidades de melhoria, conforme apêndice A. Em seguida, aplicou-se a técnica de grupo nominal e matriz de priorização de problemas, a qual apresentou como prioridade a melhoria no processo de identificação do paciente.

Etapa 2: Análise do problema de qualidade

Após a seleção da oportunidade de melhoria, houve a necessidade de identificar as possíveis causas das falhas na identificação do paciente, para dar

seguimento ao planejamento da melhoria pretendida. Foram utilizados o *brainstorming* e o diagrama de causa e efeito (*Ishikawa)* (APÊNDICE B), tendo sido possível levantar as possíveis causas para o problema e, em seguida, classificá-las.

As causas principais foram agrupadas e determinadas em seis categorias genéricas: pessoas, processos, equipamentos, materiais, usuários e métodos.

Etapa 3: Construção de critérios para avaliar a qualidade

Critérios podem ser compreendidos como ferramentas ou referências com as quais podemos mensurar a qualidade, assim como aspectos essenciais e relevantes dos cuidados de saúde em torno do problema. Quando avaliamos sua presença, ausência ou, ainda, seu grau de cumprimento, é possível medir diferentes níveis de qualidade (SATURNO, 2015).

Após análise das causas, baseando-se em instrumento disponível no serviço e no protocolo de identificação do Ministério da Saúde, elaboraram-se sete critérios de qualidade relacionados à identificação do paciente que foram medidos e avaliados antes e após a realização da intervenção proposta. Os critérios estão dispostos no Quadro 2.

Para que os critérios sejam interpretados do mesmo modo e para que não haja possibilidade de equívocos na compreensão por todos os avaliadores (se houver mais de um), é necessário detalhar as possíveis exceções e esclarecimentos.

Quadro 2. Critérios para avaliar a qualidade técnico-científica da identificação dos pacientes, Natal/Rio Grande do Norte, 2019

CRITÉRIOS	EXCEÇÕES	ESCLARECIMENTOS
**C1. Presença da pulseira de identificação.**	Pacientes edemaciados, com ferimentos, restrição e/ou amputação de membros.	
**C2. Dados legíveis na pulseira.**		
**C3. Pulseira com dados corretos.**		Nome completo, data de nascimento e/ou número de registro do prontuário.
**C4. Colocação no membro conforme protocolo.**	Membros edemaciados, com ferimentos, restrição e/ou amputação.	Membro superior direito.
**C5. Identificação na capa do prontuário**		Não utilizar abreviações.

**contendo, ao menos, dois identificadores.**		
**C6. Identificação em todas as folhas presentes no prontuário.**		Conter nome completo do paciente e pelo menos mais um identificador, como número de registro e/ou data de nascimento.
**C7. Identificação à beira leito.**		Identificação fixada de maneira visível a todos, contendo ao menos nome completo e data de nascimento.<br>Para a pediatria, é necessário o nome da genitora.

Fonte: própria da pesquisa.

Os critérios foram avaliados quanto à porcentagem de cumprimento, observando-se todos os pacientes internados no CCI, em novembro de 2019. Conforme os dados coletados, foi possível visualizar as fragilidades e, assim, elaborar um plano de treinamento para a equipe, baseando-se nas principais necessidades.

Para realização da coleta dos dados, inicialmente foi solicitada a autorização da instituição por meio da assinatura de uma carta de anuência formal. Em seguida, o projeto foi submetido para apreciação do Comitê de Ética em Pesquisa e, somente após a sua aprovação, deu-se início à coleta de dados.

A coleta de dados foi realizada no CCI, através de um instrumento estruturado (APÊNDICE C) para avaliação do cumprimento dos critérios. Foi dividida em três momentos: uma avaliação inicial antes da intervenção, que ocorreu em novembro de 2019; outra um mês após a capacitação, que ocorreu em outubro de 2020; e, por último, realizou-se a terceira coleta dois meses após a intervenção, em novembro de 2020.

A coleta de dados foi realizada pela mestranda. Os pacientes e/ou responsáveis foram orientados quanto aos objetivos da pesquisa, à forma de coleta de dados, benefícios, riscos e, após o aceite, foram solicitadas a leitura e assinatura do Termo de Consentimento Livre e Esclarecido (APÊNDICE D).

1. 2. 5 Desenvolvimento da intervenção

Após a primeira etapa de coleta, os dados foram analisados e utilizados como subsídio para o planejamento participativo da intervenção focada nos defeitos de qualidade identificados. A intervenção se baseou em duas linhas estratégicas: organização dos processos de trabalho e capacitação da equipe, conforme o Quadro 3, e estruturada em quatro fases, como descritas abaixo.

Etapas 4 e 5: Planejamento do estudo para avaliar o nível de qualidade e Intervenção de melhoria dirigida aos critérios mais problemáticos

Fase 1 – Recrutamento dos profissionais

Foram realizados cinco encontros com os enfermeiros do CCI nos quais foram realizadas abordagens expositivas dialogadas sobre a importância da identificação do paciente. Também foram expostos os dados da primeira avaliação, para que servissem como situação problema de partida para as demais fases.

Fase 2 – Apresentação e discussão dos documentos institucionais

Foram disponibilizados o Protocolo institucional e o Protocolo Operacional Padrão (POP) sobre a identificação correta do paciente, que haviam sido revisados anteriormente, para leitura e discussão.

Fase 3 – Capacitação em serviço

Após os encontros, os enfermeiros passaram a ser os multiplicadores do conhecimento e realizaram a capacitação em serviço de suas equipes baseados no que havia sido discutido anteriormente. O enfermeiro de cada turno utilizou como norteador teórico o POP para discussão com a equipe. As capacitações ocorreram durante o período de três semanas, em todos os turnos, para alcançar o maior número de profissionais possível.

Fase 4 – Recursos de identificação

Paralelamente às atividades educativas, foi enviada à direção uma proposta para substituição das pulseiras de identificação, pois as que estavam em uso não atendiam à necessidade, e aquisição dos *displays* para identificação à beira do leito para padronização.

Quadro 3. Organização da intervenção de educação permanente a ser implementada após a primeira etapa da coleta de dados, Natal/Rio Grande do Norte, 2019

ORGANIZAÇÃO DOS PROCESSOS DE TRABALHO	CAPACITAÇÃO DA EQUIPE
Atualização do protocolo de identificação da instituição;<br>Revisão do Protocolo Operacional Padrão (POP);<br>Proposta de substituição de pulseiras identificadoras;<br>Repaginação das placas de identificação à beira do leito, com reconfiguração do *layout* dos *displays;*<br>Emissão das placas de identificação via prontuário eletrônico.	Capacitações, em horários alternados, com os enfermeiros do CCI, expondo os eventos adversos relacionados ao processo de identificação do paciente;<br>Treinamento do Protocolo Operacional Padrão junto aos enfermeiros, os quais ficaram responsáveis por capacitar e implementar o processo para a equipe.

Fonte: própria da pesquisa.

As intervenções educativas foram realizadas em parceria com Núcleo de Educação Permanente (NEP) da instituição.

Após as intervenções, foi realizada a segunda etapa da coleta dos dados, com o intuito de verificar a ocorrência de mudanças positivas que demonstrassem melhorias, com vistas à qualidade da identificação do paciente, além da segurança e melhoria da assistência.

1. 2. 6 Análise dos dados

Realizou-se análise descritiva das variáveis por meio de distribuições de frequências absolutas e relativas (%). Na avaliação da conformidade por critérios analisados, aplicou-se o teste de Qui-Quadrado. Para os testes estatísticos aplicados, considerou-se o nível de significância de 5%.

O teste Qui-Quadrado permite avaliar se as variáveis estão relacionadas com determinado nível de significância.

Hipóteses a serem testadas:

Hipótese Nula (H_0): A intervenção aplicada não foi efetiva para o cumprimento dos critérios (as variáveis são independentes).

Hipótese Alternativa (H_1): A intervenção aplicada foi efetiva para o cumprimento dos critérios (as variáveis são dependentes).

A hipótese de nulidade pode ser testada por:

$$\chi^2_{cal} = \sum_{i=1}^{k} \frac{(O_i - E_i)^2}{E_i} \approx \chi^2_{(k-1)}$$

Onde O_i = n° de casos observados classificados na categoria i;

E_i = n° de casos esperados classificados na categoria i, sob H_0.

Assim, rejeitamos H_0, se $\chi^2 cal \geq \chi^2$ (k – 1, α), ou se a probabilidade associada à ocorrência, sob H_0, do valor obtido do χ^2cal com (k - 1) g.l. não superar o valor de alfa, ou seja, P [χ^2k - 1 $\geq \chi^2$cal] for significativo (menor que alfa).

g.l: Graus de Liberdade;

α: Nível de significância.

Para analisar o efeito da intervenção, foram estimados os valores de melhoria absoluta e relativa e a significação estatística pelo teste do valor de z ($p < 0,05$).

Os dados de não cumprimento das avaliações foram analisados utilizando um gráfico antes e depois de Pareto, a fim de visualizar a melhoria e para priorizar critérios de qualidade.

A avaliação promovida pelo Gráfico de Pareto para os resultados de não conformidade utiliza dois eixos verticais (o da esquerda para o número absoluto de casos de não cumprimento; e a o da direita para as frequências relativas correspondentes calculadas como percentual em relação ao número total de casos de não conformidade na avaliação), enquanto que o eixo horizontal é usado para um gráfico de barras dos diferentes critérios avaliados, que ordenou, a partir do número, de mais para menos frequente de não conformidades (SATURNO, 2017).

O estudo seguiu os princípios éticos de pesquisa envolvendo seres humanos, conforme a Resolução 466/12 do Conselho Nacional de Saúde (CNS), e foi aprovado pelo Comitê de Ética em Pesquisa do Hospital Universitário Onofre Lopes (HUOL/UFRN) sob o Parecer número 3.540.139 e CAAE 18537619.9.0000.5292 (BRASIL, 2012).

1. 3 Resultados

Serão apresentados os resultados das avaliações, a saber: avaliação inicial (antes da intervenção – novembro/2019); 1ª reavaliação (pós-intervenção – outubro/2020); e a 2ª reavaliação (pós-intervenção – novembro/2020).

1. 3. 1 Avaliação inicial

A primeira avaliação evidenciou níveis variáveis de cumprimento dos critérios. Dentre os 52 pacientes avaliados, os critérios que apresentaram menor cumprimento foram o C2 (dados legíveis na pulseira de identificação) e C3 (dados da pulseira de identificação corretos), que atingiram apenas 32%. Ainda se destaca o critério C4 (colocação da pulseira no membro conforme protocolo), que obteve somente 17% de conformidade.

No que se refere à identificação no prontuário, o critério C6 (identificação em todas as folhas presentes no prontuário) atingiu apenas 25% de conformidade, conforme a tabela abaixo.

Tabela 1. Avaliação inicial do cumprimento de conformidade e não conformidade por critério analisado, Natal/Rio Grande do Norte, 2019

Critério	Avaliação inicial	
	**Conforme**	**Não conforme**
**C1**	50,00% (n=26)	50,00% (n=26)
**C2**	32,69% (n=17)	67,31% (n=35)
**C3**	32,69% (n=17)	67,31% (n=35)
**C4**	17,31% (n=9)	82,69% (n=43)
**C5**	98,08% (n=51)	1,92% (n=1)
**C6**	25,00% (n=13)	75,00% (n=39)
**C7**	59,62% (n=31)	40,38% (n=21)
**Total**	**100,00% (n=52)**	

1. 3. 2 Reavaliação após intervenção (1ª e 2ª reavaliações)

De acordo com a Tabela 2 abaixo, pode-se inferir que a qualidade do processo de identificação apresentou melhoria significativa nos valores referentes ao cumprimento das conformidades, ao compararmos os resultados pré e pós-intervenção.

Tabela 2. Avaliação e reavaliação do cumprimento das conformidades e não conformidades por critério analisado, HRG, Natal/Rio Grande do Norte, Brasil, 2019-2020

Critério	Avaliação inicial		1ª Reavaliação		2ª Reavaliação		Valor-p
	Conforme	Não conforme	Conforme	Não conforme	Conforme	Não conforme	
**C1**	50,00% (n=26)	50,00% (n=26)	85,71% (n=42)	14,29% (n=7)	73,08% (n=38)	26,92% (n=14)	<0,001
**C2**	32,69% (n=17)	67,31% (n=35)	73,47% (n=36)	26,53% (n=13)	59,62% (n=31)	40,38% (n=21)	<0,001
**C3**	32,69% (n=17)	67,31% (n=35)	73,47% (n=36)	26,53% (n=13)	59,62% (n=31)	40,38% (n=21)	<0,001
**C4**	17,31% (n=9)	82,69% (n=43)	63,27% (n=31)	36,73% (n=18)	53,85% (n=28)	46,15% (n=24)	<0,001
**C5**	98,08% (n=51)	1,92% (n=1)	100,00% (n=49)	---	98,08% (n=51)	1,92% (n=1)	0,620
**C6**	25,00% (n=13)	75,00% (n=39)	73,47% (n=36)	26,53% (n=13)	69,23% (n=36)	30,77% (n=16)	<0,001
**C7**	59,62% (n=31)	40,38% (n=21)	77,55% (n=38)	22,45% (n=11)	73,08% (n=38)	26,92% (n=14)	0,121
**Total**	**100,00% (n=52)**		**100,00% (n=49)**		**100,00% (n=52)**		

Fonte: Da própria pesquisa

Ao aplicar o teste Qui-Quadrado (***X^2***), para um nível de significância de 5%, obtiveram-se evidências de diferença estatística significativa das avaliações nos critérios C1, C2, C3, C4 e C6. Nas quais pode-se observar maior percentual de conformidade nos respectivos critérios na 1ª e 2ª reavaliação, comparados com avaliação inicial.

O critério C5 – identificação na capa do prontuário – já apresentava um alto cumprimento desde a avaliação inicial e o manteve nas demais, logo, não houve significância estatística. Porém o critério C7 – identificação à beira do leito –, apesar de ter havido melhoria absoluta, não atingiu o nível de significância.

1. 3. 3 Oportunidades para melhoria

Ao avaliar as oportunidades de melhoria identificadas após a primeira avaliação no gráfico de Pareto (Figura 2), observa-se que os poucos vitais foram a colocação da pulseira no membro conforme o protocolo institucional (C4), identificação em todas as folhas do prontuário (C6), dados legíveis e corretos na pulseira de identificação (critérios C2 e C3, respectivamente), acumulando uma frequência de 76% de todos os defeitos de qualidade.

Também se observou através do gráfico de Pareto - antes-depois que, na frequência absoluta, foram 200 não cumprimentos na avaliação inicial, e 75 na 1ª reavaliação, o que representa uma redução de 125 defeitos de qualidade. E, apesar de ter havido um aumento para 111 na 2ª reavaliação, percebe-se que houve sustentabilidade na melhoria alcançada.

Nos resultados após a 1ª e 2ª reavaliações, os mesmos critérios prevaleceram como os poucos vitais, porém, com os valores absolutos consideravelmente inferiores à primeira, o que ilustra a efetividade do estudo e a melhoria alcançada. A

demonstração dos resultados, de modo agrupado, das três avaliações permite ressaltar a melhoria progressiva e a consequente diminuição das não conformidades.

Figura 2. Diagrama de Pareto evidenciando a frequência de não conformidades dos critérios de qualidade, Natal/Rio Grande do Norte, 2020

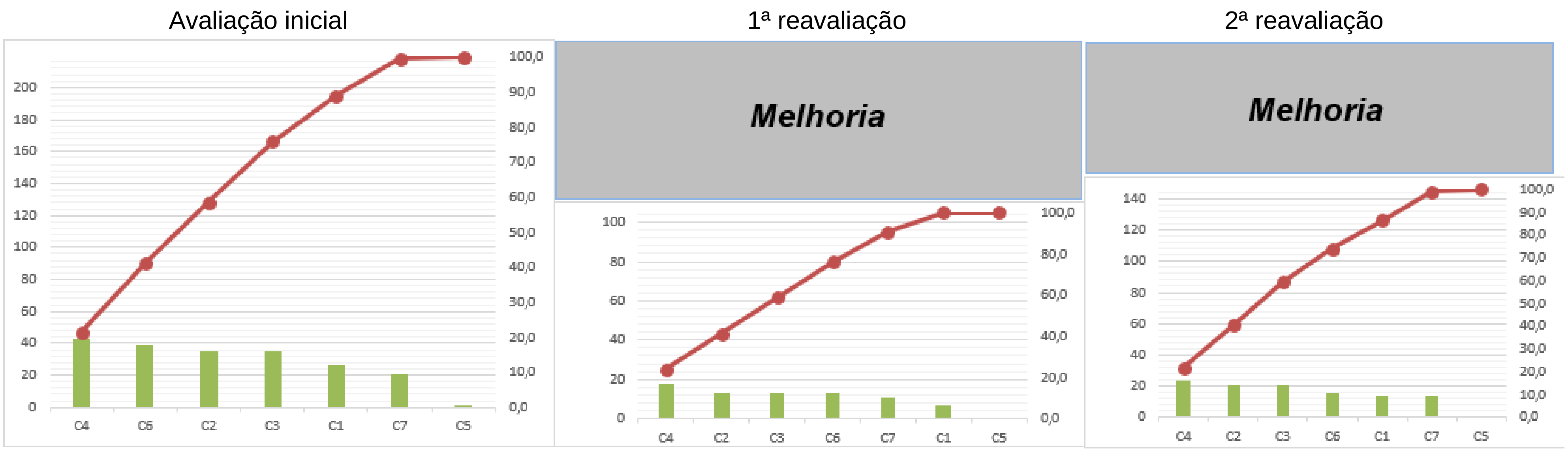

Fonte: Dados da pesquisa, 2020.

1. 4 Discussão

Em relação ao critério C1 – presença da pulseira de identificação –, o presente estudo identificou na avaliação inicial que 50% dos pacientes possuíam a pulseira, entretanto, apenas 32% apresentavam os dados legíveis e, consequentemente, corretos (critério 2 e 3). Após a intervenção, na 1ª reavaliação 85% dos pacientes estavam com pulseira e, destas, 73% encontravam-se com os dados legíveis e corretos.

Um estudo realizado em três UTI de um hospital de grande porte no Estado da Bahia mostrou que apenas 59% dos pacientes estavam em uso da pulseira de identificação e, destes, em apenas 37,6% os dados estavam legíveis, resultados semelhantes aos encontrados na avaliação inicial (TRINDADE *et al.,* 2018).

Já, nos achados de um estudo realizado na UTI de um hospital no Estado do Rio de Janeiro, constatou-se que 96% dos pacientes estavam com a pulseira de identificação, resultado superior ao encontrado no presente estudo. Soma-se a isso estudo descritivo realizado na Turquia, que evidenciou uma taxa de 84,5% de pacientes com identificados com pulseira (CENGIZ; CELIK; HIKMET, 2016); (MACEDO *et al.,* 2017).

Segundo Hoffmeister e Moura (2015), a identificação do paciente deve atingir 100% dos pacientes, pois trata-se de uma etapa importante que antecede todos os cuidados prestados pela equipe de saúde. Para tanto, observa-se que o presente estudo apenas obteve melhores resultados após a intervenção, e isto propõe que a estratégia educativa é um importante componente na mitigação dos incidentes relacionados à identificação do paciente.

Outro aspecto relevante foi identificado em um estudo que avaliou a percepção dos pacientes com relação à pulseira de identificação, e o resultado evidenciou que nenhum profissional havia colocado a pulseira e outros afirmaram que receberam na admissão, porém guardaram na gaveta, por desconsiderar a importância de tal dispositivo. Assim, fica patente que a equipe de saúde tem papel crucial na identificação e na observação do estado de conservação da pulseira (SILVA *et. al*., 2019).

A ausência da identificação do paciente é apontada por lacunas quanto à compreensão do paciente sobre a necessidade da pulseira, profissionais que atuem para manter e seguir as rotinas de segurança do paciente e educação permanente

para a equipe de saúde. Ainda há fatores relacionados ao estado do paciente, tais como sudorese excessiva, edema nos membros, excesso de dispositivos, amputações de membros e baixo nível de consciência (TRES *et. al*., 2016; SILVA *et. al*., 2019).

Nesse viés, o paciente deve ser orientado sobre a importância da utilização das pulseiras de identificação, assim como a recomendação da não remoção desta até a alta hospitalar. Deste modo, quando as pulseiras estiverem ilegíveis ou desgastadas, estas devem ser prontamente substituídas (CENGIZ; CELIK; HIKMET, 2016).

O uso da pulseira de identificação deve ser considerado um indicador relevante para a qualidade dentro dos serviços de saúde, que proporciona maior segurança para o paciente e minimiza o risco da ocorrência de incidentes de segurança relacionados à identificação. Todavia, vale salientar que apenas a presença da pulseira, por si só, não possibilita maior segurança, é necessária a checagem antes da realização de qualquer procedimento (TASE *et al.,* 2015; TRINDADE *et al.,* 2018)

Ainda nesse contexto, destaca-se como fragilidade para o alinhamento dos processos a dificuldade para identificação na UTI pediátrica, pela pouca flexibilidade das pulseiras, limitando seu uso. Ao avaliar os dados por UTI, não foi possível observar melhoria, visto que na primeira avaliação 20% dos pacientes possuíam pulseira de identificação e, na segunda e terceira avaliação, atingiram 30% e 20%, respectivamente. Todavia, após apresentação dos dados à diretoria, foi realizada aquisição de pulseiras específicas para o público infantil.

Em estudo realizado em três hospitais no Sul do Brasil, que avaliou as práticas de identificação do paciente em uma UTI pediátrica, não havia nenhuma identificação por meio de pulseira e 98% dos leitos estavam identificados através de placas à beira do leito. Também foram encontradas falhas na identificação no prontuário, resultado semelhante ao do presente estudo, demonstrando a vulnerabilidade da segurança para o paciente (BERNAL *et al.,* 2018).

No que se refere aos critérios C2 e C3 – dados legíveis e corretos na pulseira –, as pulseiras apresentavam as etiquetas de identificação apagadas, o que impossibilitou a legibilidade dos dados, bem como a confirmação dos mesmos, atingindo, assim, apenas 32% de conformidade.

Para minimizar os riscos e evitar incidentes, como falhas durante a administração de medicamentos, equívocos no gerenciamento e infusão de hemocomponentes, procedimentos cirúrgicos em pacientes incorretos, é importante

destacar que, durante a permanência do paciente no ambiente hospitalar, seja fornecida identificação com, pelo menos, duas informações relevantes: nome completo, data de nascimento, e/ou número de registro. Além disso, destaca-se que, para minimizar-se a ocorrência de erros na identificação do paciente, deve-se checar a identificação do paciente antes da realização de todo e qualquer procedimento em âmbito hospitalar (JEON *et al*., 2019; FERGUSON *et al.*, 2019).

Diferente dos dados encontrados, pesquisa feita em um hospital universitário da Região Sul do Brasil constatou que apenas 0,81% apresentaram algum tipo de dano que inviabilizou a leitura e confirmação dos dados. Isso se deve, possivelmente, ao material com o qual a pulseira era confeccionada (HOFFMEISTER; MOURA, 2015).

O presente estudo também identificou desconhecimento dos profissionais quanto ao protocolo institucional e a não adesão às boas práticas relacionadas à identificação, visto que, além dos resultados dos outros critérios, os que apresentaram maior número de não conformidades foram a não colocação da pulseira de identificação no membro conforme o protocolo institucional (C4) e a não identificação nas folhas de prontuários (C6), correspondendo a 21,5% e 19,5%, respectivamente.

Dados do estudo realizado em três hospitais no Rio Grande do Norte para verificar a conformidade na identificação do paciente nos registros em prontuário corroboram com os dados encontrados, evidenciando a fragilidade no processo (ALVES *et al*., 2018).

Um estudo brasileiro que realizou a aplicação de um ciclo de melhoria, também com o objetivo de melhorar o processo de identificação, em dois hospitais oncológicos, detectou apenas 25% de cumprimento no critério referente à identificação nas folhas dos prontuários, corroborando com os dados encontrados inicialmente no estudo em questão (GOMES NETO JUNIOR, 2016).

Em relação ao critério C7 – uso de placas de identificação à beira do leito –, os valores na avaliação pré-intervenção foram de 59% de conformidade. Porém, é válido destacar que não havia na instituição uma padronização para estas placas e cada unidade do CCI havia desenvolvido uma de modo individual. Após a intervenção, a taxa conformidade aumentou para 77%.

Noutro estudo, realizado em seis UTIs de uma instituição hospitalar no Estado de São Paulo, pôde-se observar que 99,47% dos leitos possuíam identificação, número superior ao encontrado no estudo em questão. Dessa forma, nota-se que

ainda é necessária a busca pela melhoria relacionada à segurança do paciente (ZAMPOLLO *et al.,* 2018).

É preciso destacar que a identificação à beira do leito não deve ser o único critério para verificar a identificação do paciente, isso deve ocorrer de modo sequencial à checagem da pulseira de identificação, conforme orientação estabelecida no protocolo do Ministério da Saúde (LEMOS; CUNHA, 2017).

Percebe-se que a segurança do paciente é um eixo transversal que perpassa todos os níveis de complexidade na área da saúde e que influencia de modo direto na qualidade da assistência. Nesse sentido, a identificação correta do paciente torna-se um desafio por seu papel essencial para o desenvolvimento das boas práticas dentro das instituições de saúde (PINTO; SANTOS, 2020; SILVA *et al.*, 2020).

Destaca-se, ainda, que estudos demonstram a eficácia da educação permanente para a melhoria da segurança do paciente. Pesquisa realizada na Irlanda adotou um programa educativo, visando à segurança na administração de medicamentos. Dessa forma, adotou-se uma estratégia composta por três fases. Na primeira, análise de documentos, foram analisadas as diretrizes da organização e as políticas para o gerenciamento de medicamentos. Na segunda fase, foram realizadas entrevistas com especialistas na área de segurança do paciente, a fim de refinar o conhecimento dos profissionais de saúde sobre a temática. Por fim, a fase três envolveu um estudo de caso, e posterior discussão. O estudo evidenciou que a intervenção possibilitou o aumento do raciocínio clínico dos profissionais, atenuando o número de erros na administração de medicamentos (BROWNE; HANNIGAN; HARDEN, 2021).

Nesse viés, vale salientar que a interdisciplinaridade possui grande importância para a efetividade da educação permanente em saúde. No entanto, esta ainda se configura como um desafio, devido à resistência de algumas categorias profissionais, pois a fragmentação do trabalho prejudica a ação interdisciplinar, refletindo negativamente na mudança da cultura de segurança (KOERICH; ERDMANN; LANZONI, 2020).

O tempo decorrido entre a solicitação e a disponibilização dos *displays* das placas de identificação à beira do leito pela instituição, bem como a substituição das pulseiras de identificação configuraram-se em limitações ao presente estudo. Outra limitação foi a ausência de unanimidade na adesão às novas rotinas estabelecidas por parte dos profissionais das UTI.

1. 5 Conclusões

A busca pela melhoria nos processos de segurança deve ser desenvolvida de modo contínuo e estruturado, embasada nas boas práticas. Através deste estudo, foi possível melhorar o processo de identificação do paciente na instituição em questão, demonstrando a eficácia do ciclo de melhoria. Estabelecer rotinas para um cuidado seguro demanda investimento nas atividades educativas, o que irá contribuir para o desenvolvimento e estabelecimento da cultura de segurança.

O presente estudo evidenciou que o ciclo de melhoria realizado com o objetivo de melhorar a qualidade do processo de identificação do paciente mostrou-se efetivo. Foi possível envolver as partes interessadas, estimular a participação da equipe assistencial e melhorar os processos organizacionais relacionados à temática.

A avaliação inicial evidenciou fragilidades e a necessidade de uma intervenção com foco em atividades educativas e reestruturação dos processos institucionais relacionados à identificação do paciente. Nesse sentido, é possível destacar o fortalecimento do Núcleo de Segurança do Paciente e do Núcleo de Educação Permanente, que conseguiram avigorar o processo e sensibilizar a equipe.

Faz-se necessário destacar que as atividades relacionadas à educação foram essenciais para difundir o protocolo institucional e sensibilizar os profissionais para implementação e adesão ao processo. O apoio das lideranças também foi essencial.

Apesar da melhoria perceptível, é necessário destacar que para a sustentabilidade do processo é preciso realizar o monitoramento periódico, para que seja mantida a melhoria alcançada e desenvolvidos os pontos que apresentarem fragilidades.

REFERÊNCIAS

ALVES, Kisna Yasmin Andrade *et al.* Identificação do paciente nos registros dos profissionais de saúde. **Acta Paulista de Enfermagem**, v. 31, n.1, 2018. p. 79-86. Disponível em: https://www.scielo.br/scielo.php?pid=S0103-21002018000100079&script=sci_arttext. Acesso em: 12 de fev. 2019

BERNAL, Suelen Cristina Zandonadi *et al.* Práticas de identificação do paciente em unidade de terapia intensiva pediátrica. **Cogitare Enfermagem**, v. 23, n. 3, 2018. Disponível em: https://www.hcrp.usp.br/revistaqualidade/edicaoselecionada.aspx?Edicao=4. Acesso em: 14 de jan. 2021

BRASIL. Ministério da Saúde. **Resolução nº 466, de 12 de Dezembro de 2012**. Aprova as diretrizes e normas regulamentadoras de pesquisas envolvendo seres humanos. Brasília, DF: Ministério da Saúde, 2012. Disponível em: bvsms.saude.gov.br/bvs/saudelegis/cns/.../res0466_12_12_2012.ht... Acesso em: 16 fev. 2019.

_______. Ministério da Saúde. **Resolução n° 36, de 25 de julho de 2013.** Institui ações para a segurança do paciente em serviços de saúde e dá outras providências. Brasília, 2013. Disponível em: http://bvsms.saude.gov.br/bvs/saudelegis/anvisa/2013/rdc0036_25_07_2013.html. Acesso em: 12 jan. 2021.

_______. Ministério da Saúde. **Incidentes relacionados à assistência à saúde**. Resultados das notificações realizadas no Notivisa - Brasil, setembro de 2019 a agosto de 2020. Brasília, 2020. Disponível em: https://www20.anvisa.gov.br/segurancadopaciente/index.php/publicacoes/category/eventos-adversos. Acesso em: 18 de nov. 2020.

BROWNE, Freda.; HANNIGAN, Ben.; HARDEN, Jane. A realist evaluation of a safe medication administration education programme. **Nurse Education Today**, Edimburgo, v. 97, n. 2020, 2021. p. 1-9. Disponível em: https://www.sciencedirect.com/science/article/pii/S0260691720315355. Acesso em: 23 fev 2021.

CENGIZ, Canan.; CELIK, Yusuf.; HIKMET, Neset. Evaluation of patient wristbands and patient identification process in a training hospital in Turkey. **International Journal of Health Care Quality Assurance, Bardford,** v. 29, n. 8, 2016. p. 820-834. Disponível em: https://pubmed.ncbi.nlm.nih.gov/27671418/. Acesso em: 22 fev. 2021.

FERGUSON, Caleb. et al. The wicked problem of patient misidentification: How could the technological revolution help address patient safety?. **Journal of Clinical Nursing**, Boston, v. 28, 2019. p. 2365-2368. Disponível em: https://onlinelibrary.wiley.com/doi/full/10.1111/jocn.14848. Acesso em: 22 fev. 2021.

GIRARDI, Camila *et al.* Adesão à identificação do paciente em hospital universitário público. **Revista de Administração em Saúde**, v. 18, n. 70, 2018. Disponível em: https://cqh.org.br/ojs-2.4.8/index.php/ras/article/view/70. Acesso em: 12 de fev. 2019.

GOMES NETO JÚNIOR, José. **Ciclos de melhorias para uma correta identificação do paciente em hospitais oncológicos**. Dissertação (Mestrado em Gestão da Qualidade em Serviços de Saúde)- Centro de Ciências da Saúde, Universidade Federal do Rio Grande do Norte.

HOFFMEISTER, Louíse Viecili; MOURA, Gisela Maria Schebella Souto de. Uso de pulseras de identificación en pacientes internados en un hospital universitário. **Revista Latino-Americana de Enfermagem**, v. 23, n. 1, 2015. p. 36-43. Disponível em: https://www.scielo.br/pdf/rlae/v23n1/pt_0104-1169-rlae-23-01-00036.pdf. Acesso em: 22 de jan. 2021.

JEON, Byoungjun *et al*. A facial recognition mobile app for patient safety and biometric identification: design, development, and validation. **JMIR Health and Health**, Michigan, v. 7, n. 4, 2019. p. 1-11. Disponível em: https://mhealth.jmir.org/2019/4/e11472/?fbclid=IwAR03k4uFy2RM8SZvSGnoB6nbdbgETWLGznUbhS5C-Dt08tDiFTpjCxKKWU8&utm_campaign=JMIR_TrendMD_1&utm_medium=cpc&utm_source=TrendMD. Acesso em: 22 fev. 2021.

KOERICH, Cintia.; ERDMANN, Alacoque Lorenzin.; LANZONI, Gabriela Marcelino de Melo. Professional interaction in management of the triad: Permanent Education in Health, patient safety and quality. **Revista Latino-Americana de Enfermagem**, Ribeirão Preto, v. 28, 2020. p. 1-10. Disponível em: https://www.scielo.br/pdf/rlae/v28/0104-1169-rlae-28-e3379.pdf. Acesso em: 23 fev. 2021.

LEMOS, Carina Salgado; CUNHA, Karinne Cristinne da Silva. O uso da identificação de pacientes em uma unidade hospitalar. **Rev. enferm. UFPE on line**, 2017. p. 130-139. Disponível em: https://pesquisa.bvsalud.org/portal/resource/pt/biblio-1031853. Acesso em: 10 de fev. 2021.

MACEDO, Mirian Carla de Souza *et al.* Identificação do paciente por pulseira eletrônica numa unidade de terapia intensiva geral adulta. **Revista de Enfermagem Referência**, n. 13, 2017.p.63-70.Disponível em: http://www.scielo.mec.pt/scielo.php?script=sci_arttext&pid=S0874-02832017000200007&lng=pt&nrm=iso. Acesso em: 16 de fev. 2019.

NEDEL, Wagner Luis; SILVEIRA, Fernando da. Os diferentes delineamentos de pesquisa e suas particularidades na terapia intensiva. **Revista Brasileira de Terapia Intensiva**, v. 28, n. 3, 2016. p. 256-260. Disponível em: <http://www.scielo.br/scielo.php?script=sci_arttext&pid=S0103-507X2016000300256&lng=en&nrm=iso. Acesso em: 16 de fev. 2019.

PINTO, Adriana Avanzi Marques; DOS SANTOS, Francielle Thays. Segurança do paciente: concepção e implantação da cultura de qualidade. **Brazilian Journal of Development**, v. 6, n. 3, 2020. p. 9796-9809.

Disponível: https://www.brazilianjournals.com/index.php/BRJD/article/view/7302. Acesso em: 13 de fev. 2020.

PORTELA, Margareth Crisóstomo *et al.* Republicado: Como estudar as intervenções de melhoria: uma breve visão geral dos possíveis tipos de estudo. **Jornal médico de pós-graduação** , v. 91, n. 1076, 2015. p. 343-354. Disponível em: https://qualitysafety.bmj.com/content/24/5/325.info. Acesso em: 16 de fev. 2019.

SAFETY, WHO Patient *et al.* **Conceptual framework for the international classification for patient safety version 1.1: final technical report January 2009**. World Health Organization, 2009. Disponível em: https://www.who.int/patientsafety/implementation/taxonomy/ICPS-report/en/Acesso em: 23 de out. 2019.

SATURNO-HERNÁNDEZ, Pedro Jesus -Hernandez. **Métodos y herramientas para larealización de ciclos de mejora de lacalidad em servicios de salud**. 1. ed. Cuernavaca, México: Instituto Nacional de Salud Pública, 2015. Disponível em: https://www.saludpublica.mx/index.php/spm/article/view/7568/10846. Acesso em: 16 de fev. 2019.

SATURNO HERNÁNDEZ, Pedro Jesus. Análise e apresentação dos dados de uma avaliação: unidade temática 7. In: SATURNO HERNÁNDEZ, Pedro Jesus *et al.* Atividades básicas para melhoria contínua: métodos e instrumentos para realizar o ciclo de melhoria: módulo II. Tradução de Zenewton André da Silva Gama; Organização de Pedro Jesus Saturno Hernández. Natal: SEDIS-UFRN, 2017.

SILVA, Mariana Meneses Rocha Pereira *et al*. Análise da adesão da identificação do paciente no setor de clínica médica. **Revista Enfermagem Atual In Derme**, v. 87, n. 25, 2019. Disponível em: https://revistaenfermagematual.com.br/index.php/revista/article/view/188/90. Acesso em: 14 de jan. 2021.

SILVA, João Miguel Almeida Ventura *et al*. Identificação do paciente como estratégia de segurança. **Rev. enferm. UFPE on line**, p. 1-11, 2020. Disponível em: https://pesquisa.bvsalud.org/portal/resource/pt/biblio-1116165. Acesso em: 12 de fev. 2021.

TASE, Terezinha Hideco; QUADRADO, Ellen Regina Sevilla; TRONCHIN, Daisy Maria Rizatto. Avaliação do risco de erro na identificação de mulheres numa maternidade pública. **Revista Brasileira de Enfermagem**, v. 71, n. 1, 2018. p. 120-125. Disponível em: http://www.scielo.br/scielo.php?script=sci_arttext&pid=S0034-71672018000100120&lng=en&nrm=iso. Acesso em: 14 de mar. 2019.

TASE, Terezinha Hideco; TRONCHIN, Daisy Maria Rizatto. Sistemas de identificação de pacientes em unidades obstétricas e a conformidade das pulseiras. **Acta Paulista de Enfermagem**, v. 28, n. 4, 2015. p. 374-380. Disponível em: https://www.scielo.br/scielo.php?pid=S0103-21002015000400014&script=sci_abstract&tlng=pt. Acesso em: 14 de abr. 2019

TRES, Daniela Patrícia et al. Qualidade da assistência e segurança do paciente: avaliação por indicadores. **Cogitare Enfermagem**, v. 21, n. 5, 2016. Disponível em: https://revistas.ufpr.br/cogitare/article/view/44938/pdf. Acesso em: 10 de jan. 2021.

TRINDADE, Thaís Venas da Costa *et al.* Segurança do paciente em unidade de terapia intensiva: uso de pulseiras de identificação. **Enfermagem Brasil**, v. 18, n. 2, 2019. p. 225-233. Disponível em: https://portalatlanticaeditora.com.br/index.php/enfermagembrasil/article/view/2309. Acesso em: 25 de set. 2020.

ZAMPOLLO, Natália *et al.* Adesão ao protocolo de identificação do paciente e medicação segura. **Rev. enferm. UFPE** on line, 2018. p. 2667-2674. Disponível em: https://www.researchgate.net/publication/334617583_Adesao_ao_protocolo_de_identificacao_do_paciente_e_medicacao_segura. Acesso em: 14 de fev. 2021.

2 APÊNDICES

2. 1 Apêndice 1

UNIVERSIDADE FEDERAL DO RIO GRANDE DO NORTE
CENTRO DE CIÊNCIAS DA SAÚDE
MESTRADO PROFISSIONAL GESTÃO DA QUALIDADE EM SERVIÇOS DE SAÚDE

Quadro 1. Matriz de priorização de problemas, Natal/Rio Grande do Norte, 2019

CRITÉRIOS DE DECISÃO					
**PROBLEMA**	**Afeta muitos pacientes?**	**Representa um risco grave à saúde?**	**A possível solução depende de esforços internos?**	**É uma solução barata?**	**Total**
1	5	5	5	3	18
2	5	4	4	4	17
3	4	3	5	5	17
4	4	4	4	4	16
5	5	4	5	4	17
6	4	2	5	5	16

Legenda:
1Melhoria no processo de identificação do paciente;
2 Protocolo de quedas;
3 Organização do agendamento de cirurgias;
4 Dispensação de medicamentos pela farmácia;
5 Realização do protocolo de cirurgia segura;
6 Dupla checagem na dispensação e administração de medicamentos.

2. 2 Apêndice 2

UNIVERSIDADE FEDERAL DO RIO GRANDE DO NORTE
CENTRO DE CIÊNCIAS DA SAÚDE
MESTRADO PROFISSIONAL GESTÃO DA QUALIDADE EM SERVIÇOS DE SAÚDE

Figura 1. Diagrama de causa e efeito aplicado à identificação dos pacientes, Natal/ Rio Grande do Norte, 2019

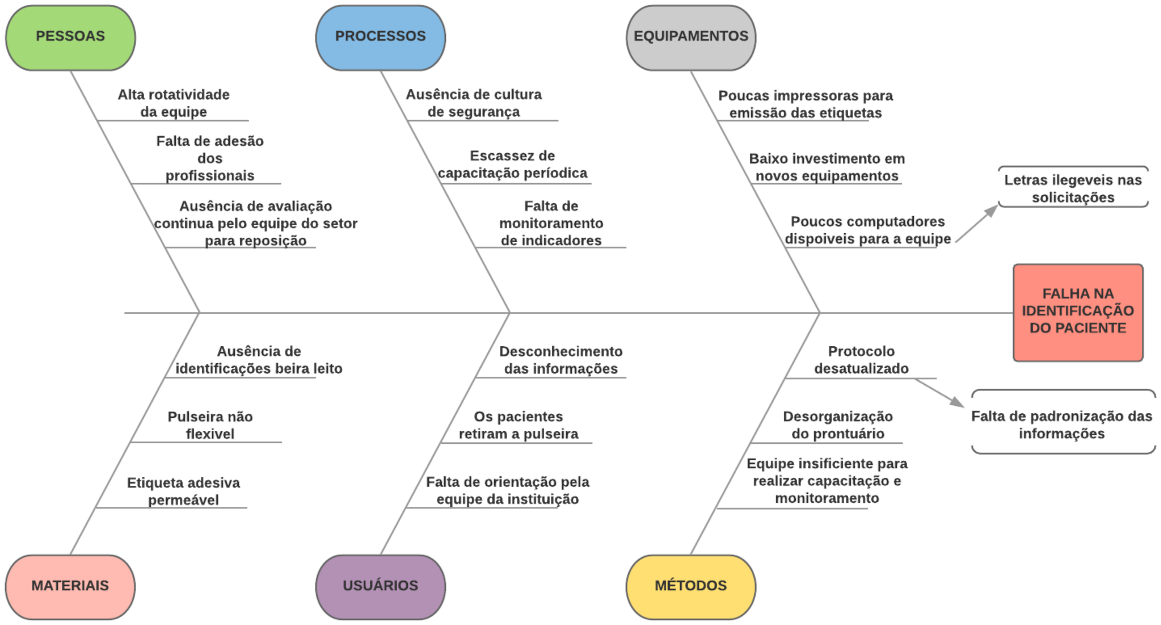

Fonte: própria da pesquisa

2. 3 Apêndice 3

UNIVERSIDADE FEDERAL DO RIO GRANDE DO NORTE
CENTRO DE CIÊNCIAS DA SAÚDE
MESTRADO PROFISSIONAL GESTÃO DA QUALIDADE EM SERVIÇOS DE SAÚDE

INSTRUMENTO DE COLETA DE DADOS

	CRITÉRIO	L01	L02	L03	L04	L05	L06	L07	L08	L09	L10	L11	L12	L13	L14	L15
1	Presença da pulseira de identificação															
2	Dados legíveis na pulseira															
3	Pulseira com dados corretos															
4	Colocação no membro conforme protocolo															
5	Identificação na capa do prontuário contendo, ao menos, dois identificadores															
6	Identificação em todas as folhas presentes no prontuário															
7	Identificação à beira do leito															

LEGENDA: S – Sim/ N – Não

2. 4 Apêndice 4

UNIVERSIDADE FEDERAL DO RIO GRANDE DO NORTE
CENTRO DE CIÊNCIAS DA SAÚDE
MESTRADO PROFISSIONAL GESTÃO DA QUALIDADE EM SERVIÇOS DE SAÚDE

TCLE para profissionais:

TERMO DE CONSENTIMENTO LIVRE E ESCLARECIDO – TCLE

Esclarecimentos

Este é um convite para você participar da pesquisa: "Identificação do Paciente em um Centro de Cuidados Intensivos: Aplicação de um Ciclo de Melhoria**"**, que tem como pesquisadora responsável a Enfermeira Maria Solange Moreira de Lima**.**

Esta pesquisa pretende avaliar o efeito da implantação de um ciclo de melhoria da qualidade no processo de identificação do paciente.

O motivo que nos leva a fazer este estudo justifica-se, uma vez que números alarmantes relacionados a erros no processo de assistência à saúde, bem como suas consequências, sejam elas temporárias ou permanentes, têm estado cada vez em maior evidência. E erros relacionados à identificação do paciente são um tema atual, que está envolvido em todo o ciclo do cuidado e diretamente vinculado à segurança do paciente.

Caso você decida participar, será preciso sua autorização. Os dados serão colhidos através de observação direta e prontuário. Não serão executados procedimentos, o risco que você corre é mínimo.

Durante a realização da pesquisa poderão ocorrer eventuais desconfortos e possíveis riscos, como algum dano ou perda do prontuário, bem como risco de exposição dos seus dados. Esses riscos poderão ser minimizados com a garantia de que apenas a pesquisadora realizará a coleta de dados e que a privacidade, sigilo das informações e preservação da sua identidade serão mantidos.

Pode acontecer um desconforto durante a intervenção, que será realizada através de encontros em grupo para exposição dialogada e atividades com metodologias ativas sobre o tema, que será minimizado ao respeitar sua privacidade, respeitando seus limites. E você terá como benefício da pesquisa aprender sobre a temática, a troca de experiências com os demais membros da equipe, além de colaborar para a pesquisa científica, contribuindo para melhorar a qualidade no processo de identificação do paciente, o declínio dos eventos adversos naqueles que são identificados erroneamente, padronizar a identificação de maneira correta, envolver e educar os profissionais que lidam com o gerenciamento do cuidado.

Em caso de algum problema que você possa ter relacionado com a pesquisa, você terá direito a assistência gratuita que será prestada pela coordenadora da pesquisa.

Durante todo o período da pesquisa, você poderá tirar suas dúvidas ligando para a enfermeira Maria Solange Moreira de Lima no telefone (84) 98828-6712 ou pelo *e-mail* solmoreira18@gmail.com.

Você tem o direito de se recusar a participar ou retirar seu consentimento, em qualquer fase da pesquisa, sem nenhum prejuízo para você.

Os dados que você irá nos fornecer serão confidenciais e serão divulgados apenas em congressos ou publicações científicas, não havendo divulgação de nenhum dado que possa identificá-lo. Esses dados serão guardados pelo pesquisador responsável por esta pesquisa em local seguro e por um período de 5 anos. Se você tiver algum gasto pela sua participação nesta pesquisa, ele será assumido pelo pesquisador e reembolsado para você.

Se você sofrer algum dano comprovadamente decorrente desta pesquisa, você será indenizado.

Qualquer dúvida sobre a ética desta pesquisa, você deverá entrar em contato com o Comitê de Ética em Pesquisa do Hospital Universitário Onofre Lopes, telefone: 3342-5003, endereço: Av. Nilo Peçanha, 620 – Petrópolis – Espaço João Machado – 1° Andar – Prédio Administrativo - CEP 59.012-300 - Nata/RN, *e-mail*: cep_huol@yahoo.com.br.

Este documento foi impresso em duas vias. Uma ficará com você e a outra com a pesquisadora responsável Maria Solange Moreira de Lima.

Consentimento Livre e Esclarecido

Após ter sido esclarecido sobre os objetivos, importância e o modo como os dados serão coletados nesta pesquisa, além de conhecer os riscos, desconfortos e benefícios que ela trará para mim e ter ficado ciente de todos os meus direitos, concordo em participar da pesquisa “Identificação do Paciente em um Centro de Cuidados Intensivos: Aplicação de um Ciclo de Melhoria**”**, e autorizo a divulgação das informações por mim fornecidas em congressos e/ou publicações científicas, desde que nenhum dado possa me identificar.

Natal, ____**/**_____**/**_________

____

Assinatura do participante da pesquisa

Impressão datiloscópica do participante

Declaração do pesquisador responsável

Como pesquisador responsável pelo estudo “Identificação do Paciente em um Centro de Cuidados Intensivos: Aplicação de um Ciclo de Melhoria**”**, declaro que assumo a inteira responsabilidade de cumprir fielmente os procedimentos metodológicos e direitos que foram esclarecidos e assegurados ao participante deste estudo, assim como manter sigilo e confidencialidade sobre a identidade do mesmo.

Declaro ainda estar ciente de que, na inobservância do compromisso ora assumido, estarei infringindo as normas e diretrizes propostas pela Resolução 466/12 do Conselho Nacional de Saúde – CNS, que regulamenta as pesquisas envolvendo o ser humano.

Natal, ____/_____/_________

____

Maria Solange Moreira de Lima
Enfermeira
COREN/RN 467.986

2. 5 Apêndice 5

UNIVERSIDADE FEDERAL DO RIO GRANDE DO NORTE
CENTRO DE CIÊNCIAS DA SAÚDE
MESTRADO PROFISSIONAL GESTÃO DA QUALIDADE EM SERVIÇOS DE SAÚDE

TCLE para maiores de idade:

TERMO DE CONSENTIMENTO LIVRE E ESCLARECIDO – TCLE

Esclarecimentos

Este é um convite para você participar da pesquisa: "Identificação do Paciente em um Centro de Cuidados Intensivos: Aplicação de um Ciclo de Melhoria", que tem como pesquisadora responsável a Enfermeira Maria Solange Moreira de Lima.

Esta pesquisa pretende avaliar o efeito da implantação de um ciclo de melhoria da qualidade no processo de identificação do paciente.

O motivo que nos leva a fazer este estudo justifica-se, uma vez que números alarmantes relacionados a erros no processo de assistência à saúde, bem como suas consequências, sejam elas temporárias ou permanentes, têm estado cada vez em maior evidência. E erros relacionados à identificação do paciente são um tema atual, que está envolvido em todo o ciclo do cuidado e diretamente vinculado à segurança do paciente.

Caso você decida participar, será preciso sua autorização. Os dados serão colhidos através do prontuário. Não serão executados procedimentos, o risco que você corre é mínimo.

Durante a realização da pesquisa poderão ocorrer eventuais desconfortos e possíveis riscos, como algum dano ou perda do prontuário, bem como risco de exposição dos seus dados. Esses riscos poderão ser minimizados com a garantia de que apenas a pesquisadora realizará a coleta de dados e que a privacidade, sigilo das informações e preservação da sua identidade serão mantidos.

Com isso, você estará colaborando para a pesquisa cientifica, contribuindo para melhorar a qualidade no processo de identificação do paciente e o declínio dos eventos adversos naqueles que são identificados erroneamente.

Em caso de algum problema que você possa ter relacionado com a pesquisa, você terá direito a assistência gratuita que será prestada pela coordenadora da pesquisa.

Durante todo o período da pesquisa você poderá tirar suas dúvidas ligando para a enfermeira Maria Solange Moreira de Lima no telefone (84) 98828-6712 ou pelo *e-mail* solmoreira18@gmail.com.

Você tem o direito de se recusar a participar ou retirar seu consentimento, em qualquer fase da pesquisa, sem nenhum prejuízo para você.

Os dados que você irá nos fornecer serão confidenciais e serão divulgados apenas em congressos ou publicações científicas, não havendo divulgação de nenhum dado que possa identificá-lo. Esses dados serão guardados pelo pesquisador responsável por esta pesquisa em local seguro

e por um período de 5 anos. Se você tiver algum gasto pela sua participação nesta pesquisa, ele será assumido pelo pesquisador e reembolsado para você.

Se você sofrer algum dano comprovadamente decorrente desta pesquisa, você será indenizado.

Qualquer dúvida sobre a ética desta pesquisa, você deverá entrar em contato com o Comitê de Ética em Pesquisa do Hospital Universitário Onofre Lopes, telefone: 3342-5003, endereço: Av. Nilo Peçanha, 620 – Petrópolis – Espaço João Machado – 1° Andar – Prédio Administrativo - CEP 59.012-300 - Nata/RN, *e-mail*: cep_huol@yahoo.com.br.

Este documento foi impresso em duas vias. Uma ficará com você e a outra com a pesquisadora responsável Maria Solange Moreira de Lima.

Consentimento Livre e Esclarecido

Após ter sido esclarecido sobre os objetivos, importância e o modo como os dados serão coletados nesta pesquisa, além de conhecer os riscos, desconfortos e benefícios que ela trará para mim e ter ficado ciente de todos os meus direitos, concordo em participar da pesquisa “Identificação do Paciente em um Centro de Cuidados Intensivos: Aplicação de um Ciclo de Melhoria”, e autorizo a divulgação das informações por mim fornecidas em congressos e/ou publicações científicas, desde que nenhum dado possa me identificar.

Natal, ____/_____/_________

____

Assinatura do participante da pesquisa

Impressão datiloscópica do participante

Declaração do pesquisador responsável

Como pesquisador responsável pelo estudo “Identificação do Paciente em um Centro de Cuidados Intensivos: Aplicação de um Ciclo de Melhoria”, declaro que assumo a inteira responsabilidade de cumprir fielmente os procedimentos metodológicos e direitos que foram esclarecidos e assegurados ao participante deste estudo, assim como manter sigilo e confidencialidade sobre a identidade do mesmo.

Declaro ainda estar ciente de que, na inobservância do compromisso ora assumido, estarei infringindo as normas e diretrizes propostas pela Resolução 466/12 do Conselho Nacional de Saúde – CNS, que regulamenta as pesquisas envolvendo o ser humano.

Natal, ____/_____/_________

____

Maria Solange Moreira de Lima
Enfermeira
COREN/RN 467.986

2. 6 Apêndice 6

UNIVERSIDADE FEDERAL DO RIO GRANDE DO NORTE
CENTRO DE CIÊNCIAS DA SAÚDE
MESTRADO PROFISSIONAL GESTÃO DA QUALIDADE EM SERVIÇOS DE SAÚDE

TCLE para responsáveis:

TERMO DE CONSENTIMENTO LIVRE E ESCLARECIDO – TCLE

Esclarecimentos

Estamos solicitando a você a autorização para que o paciente pelo qual você é responsável participe da pesquisa: "Identificação do Paciente em um Centro de Cuidados Intensivos: Aplicação de um Ciclo de Melhoria**"**, que tem como pesquisadora responsável a Enfermeira Maria Solange Moreira de Lima**.**

Esta pesquisa pretende avaliar o efeito da implantação de um ciclo de melhoria da qualidade no processo de identificação do paciente.

O motivo que nos leva a fazer este estudo justifica-se, uma vez que números alarmantes relacionados a erros no processo de assistência à saúde, bem como suas consequências, sejam elas temporárias ou permanentes, têm estado cada vez em maior evidência. E erros relacionados à identificação do paciente são um tema atual, que está envolvido em todo o ciclo do cuidado e diretamente vinculado à segurança do paciente.

Caso você concorde em autorizar a participação do paciente sob sua responsabilidade, será preciso sua autorização. Os dados serão colhidos através de observação direta e do prontuário. Não serão executados procedimentos, o risco que o paciente corre é mínimo.

Durante a realização da pesquisa poderão ocorrer eventuais desconfortos e possíveis riscos, como algum dano ou perda do prontuário, bem como risco de exposição dos dados do paciente. Esses riscos serão minimizados com a garantia de que apenas a pesquisadora realizará a coleta de dados e que a privacidade, sigilo das informações e preservação da identidade do paciente serão mantidos.

Com isso, você estará colaborando para a pesquisa cientifica, contribuindo para melhorar a qualidade no processo de identificação do paciente e o declínio dos eventos adversos naqueles que são identificados erroneamente.

Em caso de algum problema que ele(a) possa ter, relacionado com a pesquisa, ele(a) terá direito a assistência gratuita que será prestada pela coordenadora da pesquisa.

Durante todo o período da pesquisa, você poderá tirar suas dúvidas ligando para a enfermeira Maria Solange Moreira de Lima no telefone (84) 98828-6712 ou pelo *e-mail* solmoreira18@gmail.com.

Você tem o direito de recusar sua autorização, em qualquer fase da pesquisa, sem nenhum prejuízo para você e para ele(a). Os dados utilizados serão confidenciais e serão divulgados apenas em congressos ou publicações científicas, não havendo divulgação de nenhum dado que possa identificar o paciente.

Esses dados serão guardados pelo pesquisador responsável por esta pesquisa em local seguro e por um período de 5 anos.

Se você tiver algum gasto pela participação dele(a) nesta pesquisa, ele será assumido pelo pesquisador e reembolsado para você. Se ele(a) sofrer algum dano comprovadamente decorrente desta pesquisa, ele(a) será indenizado.

Qualquer dúvida sobre a ética desta pesquisa, você deverá entrar em contato com o Comitê de Ética em Pesquisa do Hospital Universitário Onofre Lopes, telefone: 3342-5003, endereço: Av. Nilo Peçanha, 620 – Petrópolis – Espaço João Machado – 1° Andar – Prédio Administrativo - CEP 59.012-300 - Nata/RN, *e-mail*: cep_huol@yahoo.com.br.

Este documento foi impresso em duas vias. Uma ficará com você e a outra com a pesquisadora responsável Maria Solange Moreira de Lima.

Consentimento Livre e Esclarecido

Eu, ____, representante legal do paciente ____, autorizo sua participação na pesquisa "Identificação do Paciente em um Centro de Cuidados Intensivos: Aplicação de um Ciclo de Melhoria".

Esta autorização foi concedida após os esclarecimentos que recebi sobre os objetivos, importância e o modo como os dados serão coletados, por ter entendido os riscos, desconfortos e benefícios que esta pesquisa pode trazer para ele(a) e também por ter compreendido todos os direitos que ele(a) terá como participante, e eu como sua representante legal. Autorizo, ainda, a publicação das informações fornecidas através do prontuário em congressos e/ou publicações científicas, desde que os dados apresentados não possam identificá-lo(a).

Natal, ____/_____/_________

____

Assinatura do participante da pesquisa

Impressão datiloscópica do participante

Declaração do pesquisador responsável

Como pesquisador responsável pelo estudo “Identificação do Paciente em um Centro de Cuidados Intensivos: Aplicação de um Ciclo de Melhoria”, declaro que assumo a inteira responsabilidade de cumprir fielmente os procedimentos metodológicos e direitos que foram esclarecidos e assegurados ao participante deste estudo, assim como manter sigilo e confidencialidade sobre a identidade do mesmo.

Declaro ainda estar ciente de que, na inobservância do compromisso ora assumido, estarei infringindo as normas e diretrizes propostas pela Resolução 466/12 do Conselho Nacional de Saúde – CNS, que regulamenta as pesquisas envolvendo o ser humano.

Natal, ____/_____/_________

____

Maria Solange Moreira de Lima
Enfermeira
COREN/RN 467.986

3 ANEXOS

3. 1 Anexo 1

UFRN - HOSPITAL UNIVERSITÁRIO ONOFRE LOPES DA UNIVERSIDADE FEDERAL DO RIO GRANDE DO NORTE - HUOL/UFRN

PARECER CONSUBSTANCIADO DO CEP

DADOS DO PROJETO DE PESQUISA

Título da Pesquisa: AVALIAÇÃO E MELHORIA NA IDENTIFICAÇÃO DO PACIENTE DE UM HOSPITAL PRIVADO DE GRANDE PORTE

Pesquisador: MARIA SOLANGE MOREIRA DE LIMA

Área Temática:

Versão: 1

CAAE: 18537619.9.0000.5292

Instituição Proponente: PROGRAMA DE PÓS-GRADUAÇÃO EM GESTÃO DA QUALIDADE EM

Patrocinador Principal: Financiamento Próprio

DADOS DO PARECER

Número do Parecer: 3.540.139

Apresentação do Projeto:

Trata-se de projeto de trabalho de Conclusão de aluno de Mestrado Profissional vinculado ao Programa de Pós-Graduação em Gestão da Qualidade em Serviços de Saúde da Universidade Federal do Rio Grande do Norte- UFRN. O estudo objetiva avaliar e melhorar a qualidade no processo de identificação do paciente em um hospital privado de grande porte do Nordeste brasileiro. Trata-se da implementação de um ciclo de melhoria interno, do tipo descritivo, observacional e de intervenção, utilizando um desenho quase-experimental, antes-depois, com abordagem quantitativa, a ser desenvolvido no período de novembro de 2019 a junho de 2020, no Hospital Rio Grande (HRG), Natal, Rio Grande do Norte (RN). Os dados serão colhidos através da observação dos prontuários durante o período de internação com previsão amostral de 200 participantes. O estudo com delineamento quase-experimental do tipo antes e depois é caracterizado pela não randomização da amostra para a implementação da intervenção (NEDEL; SILVEIRA, 2016). Os Ciclos de Melhoria da Qualidade estão compreendidos entre as três atividades de gestão dos serviços de saúde, acompanhados do planejamento e monitoramento da qualidade. Em cada avaliação será considerado o intervalo de confiança (IC) de 95% para as estimativas de conformidade. Para analisar o efeito das intervenções, serão estimados os valores de melhoria absoluta e relativa e a significação estatística pelo teste do valor de z ($p<0,05$). Os critérios

Endereço: Avenida Nilo Peçanha, 620 - Prédio Administrativo - 1º Andar - Espaço João Machado
Bairro: Petrópolis **CEP:** 59.012-300
UF: RN **Município:** NATAL
Telefone: (84)3342-5003 **Fax:** (84)3202-3941 **E-mail:** cep_huol@yahoo.com.br

Este parecer foi elaborado baseado nos documentos abaixo relacionados:

Tipo Documento	Arquivo	Postagem	Autor	Situação
Informações Básicas do Projeto	PB_INFORMAÇÕES_BÁSICAS_DO_PROJETO_1397753.pdf	06/08/2019 21:10:23		Aceito
Outros	FolhadeIdentificacaodoPesquisadorOK.pdf	06/08/2019 21:09:44	MARIA SOLANGE MOREIRA DE LIMA	Aceito
Projeto Detalhado / Brochura Investigador	Projeto_TCMCEPOK.pdf	06/08/2019 21:08:03	MARIA SOLANGE MOREIRA DE LIMA	Aceito
TCLE / Termos de Assentimento / Justificativa de Ausência	TCLE2OK.pdf	06/08/2019 21:06:26	MARIA SOLANGE MOREIRA DE LIMA	Aceito
Folha de Rosto	folhaderostook.PDF	06/08/2019 21:05:03	MARIA SOLANGE MOREIRA DE LIMA	Aceito
Outros	declaracaodenaoinicio.pdf	03/08/2019 22:38:21	MARIA SOLANGE MOREIRA DE LIMA	Aceito
Outros	termodeconfidencialidade.pdf	03/08/2019 22:35:50	MARIA SOLANGE MOREIRA DE LIMA	Aceito
Outros	cartadeanuencia.pdf	03/08/2019 22:34:08	MARIA SOLANGE MOREIRA DE LIMA	Aceito

Situação do Parecer:
Aprovado

Necessita Apreciação da CONEP:
Não

NATAL, 29 de Agosto de 2019

Assinado por:
jose diniz junior

3. 1 Anexo 2

HOSPITAL RIO GRANDE

CARTA DE ANUÊNCIA

Por ter sido informado verbalmente e por escrito sobre os objetivos e metodologia da pesquisa intitulada **Avaliação e melhoria na identificação do paciente de um hospital privado de grande porte,** coordenada pela mestranda Maria Solange Moreira de Lima e pelo Prof. Dr. Rodrigo Assis Neves Dantas, concordo em autorizar a realização de todas as etapas necessárias para a pesquisa nesta Instituição que represento.

Esta Instituição está ciente de suas corresponsabilidades como instituição coparticipante do presente projeto de pesquisa, e de seu compromisso no resguardo da segurança e bem-estar dos participantes de pesquisa nela recrutados, dispondo de infraestrutura necessária para a garantia de tal segurança e bem-estar.

Esta autorização está condicionada à aprovação prévia da pesquisa acima citada por um Comitê de Ética em Pesquisa e ao cumprimento das determinações éticas propostas na Resolução 466/12 do Conselho Nacional de Saúde – CNS e suas complementares.

O descumprimento desses condicionamentos assegura-me o direito de retirar minha anuência a qualquer momento da pesquisa.

Natal, 08 de Julho de 2019.

Luiz Roberto Leite Fonseca
Diretor Geral

(84) 4009-1000
